# A novel ER membrane protein Ehg1/May24 plays a critical role in maintaining multiple nutrient permeases in yeast under high-pressure perturbation

**DOI:** 10.1038/s41598-019-54925-1

**Published:** 2019-12-04

**Authors:** Goyu Kurosaka, Satoshi Uemura, Takahiro Mochizuki, Yuri Kozaki, Akiko Hozumi, Sayuri Suwa, Ryoga Ishii, Yusuke Kato, Saki Imura, Natsuho Ishida, Yoichi Noda, Fumiyoshi Abe

**Affiliations:** 1 Department of Chemistry and Biological Science, College of Science and Engineering, Aoyama Gakuin University, Sagamihara, Japan; 2 Division of Medical Biochemistry, Faculty of Medicine, Tohoku Medical and Pharmaceutical University, Sendai, Japan; 3 Collaborative Research Institute for Innovative Microbiology, Department of Biotechnology, The University of Tokyo, Tokyo, Japan

**Keywords:** Cell growth, Cellular microbiology, Molecular biology

## Abstract

Previously, we isolated 84 deletion mutants in *Saccharomyces cerevisiae* auxotrophic background that exhibited hypersensitive growth under high hydrostatic pressure and/or low temperature. Here, we observed that 24 deletion mutants were rescued by the introduction of four plasmids (*LEU2, HIS3, LYS2*, and *URA3*) together to grow at 25 MPa, thereby suggesting close links between the genes and nutrient uptake. Most of the highly ranked genes were poorly characterized, including *MAY24/YPR*1*53W*. May24 appeared to be localized in the endoplasmic reticulum (ER) membrane. Therefore, we designated this gene as *EHG* (ER-associated high-pressure growth gene) *1*. Deletion of *EHG1* led to reduced nutrient transport rates and decreases in the nutrient permease levels at 25 MPa. These results suggest that Ehg1 is required for the stability and functionality of the permeases under high pressure. Ehg1 physically interacted with nutrient permeases Hip1, Bap2, and Fur4; however, alanine substitutions for Pro17, Phe19, and Pro20, which were highly conserved among Ehg1 homologues in various yeast species, eliminated interactions with the permeases as well as the high-pressure growth ability. By functioning as a novel chaperone that facilitated coping with high-pressure-induced perturbations, Ehg1 could exert a stabilizing effect on nutrient permeases when they are present in the ER.

## Introduction

Pressure and temperature are thermodynamic parameters that describe the Gibbs free energy changes in chemical reactions, as well as limiting the growth and survival of organisms^1–4^. Cold-sensitive mutants have been used to analyze the assembly of macromolecules *in vivo*. Several *Saccharomyces cerevisiae* mutants with ribosomal subunit assembly defects have been isolated by screening various cold-sensitive strains^5,6^. Cells that express a mutant allele of *α*-tubulin, *tub1-729*, are cold sensitive, and they are arrested as large-budded cells with microtubule defects^7^. High hydrostatic pressure in the range of several hundred MPa (atmospheric pressure is nearly equal to 0.1 MPa = 1 bar = 0.9869 atm = 1.0197 kg of force/cm^2^; to avoid confusion, MPa is used throughout) can inactivate or sterilize microorganisms by denaturing intracellular proteins, thereby leading to the formation of irreversible protein aggregates or the disruption of cellular membranes^8,9^. Pressure in the range of several dozen MPa does not lead readily to cell death, but it can have deleterious effects on the growth of organisms that are adapted to atmospheric pressure^10,11^.

Increasing hydrostatic pressure has an effect that is analogous to decreasing temperature in terms of increasing the order and decreasing the fluidity of lipid membranes^1,12,13^. Little is known about pressure effects on membrane proteins, however, some groups have described interesting effects of high pressure on enzymatic catalysis in membrane proteins^14–18^. In *S. cerevisiae*, the growth of tryptophan auxotrophic strains (e.g., YPH499) is highly sensitive to high pressure (>15 MPa) and low temperature (10–15 °C), whereas tryptophan prototrophic strains (e.g., BY4742) or a tryptophan auxotrophic strain over-expressing tryptophan permease Tat2 can grow efficiently under both conditions^19^. The high-pressure and low-temperature sensitivity of tryptophan auxotrophic strains is due to the vulnerability of tryptophan uptake via the permeases Tat1 and Tat2^20,21^ under high pressure and low temperature, where the fluidity of the biological membranes is significantly decreased^19,22^. Thus, when genetically exploring the effects of high pressure on intracellular biological processes other than tryptophan uptake, a tryptophan prototrophic strain should be used as a parental strain.

To further determine the critical pathways related to high-pressure and low-temperature adaptation, we screened the yeast deletion library where 4,828 individual non-essential genes were disrupted in a tryptophan prototrophic BY4742 background (*leu2 his3 lys2 ura3*)^23^. Our screening identified 84 genes, including 75 genes required for high-pressure growth and 57 genes required for low-temperature growth, with a substantial overlap of 48 genes and 12 poorly characterized genes^24^, thereby obtaining unique insights into the links between genes and their functions through work and thermal energy. In addition to tryptophan biosynthetic genes (*ARO1*, *ARO2*, *TRP1, TRP2*, *TRP4*, and *TRP5*), we identified *HOM3*, *THR4*, *SER1*, *ACO1*, and *CAF17* as genes responsible for high-pressure growth, where their deletion resulted in auxotrophy for the corresponding amino acids. This clearly indicates that the uptake of amino acids via membrane permeases is generally compromised by high hydrostatic pressure and low temperature. Functional genomic, proteomic and metabolomic studies over the last 10 years have revealed that nutrient auxotrophies have clear impacts on yeast physiology, conferring slower growth rates, stress sensitivity, or altered patterns of gene expression^25–27^. Auxotrophic mutations reduce tolerance to acetic acid^28^ or high concentrations of ethanol^29^. A recent study compared the genome-wide fitness profiles of prototrophic and auxotrophic collections under diverse drug and environmental conditions in order to systematically assess the impact of auxotrophies^30^. These findings prompted us to re-analyze the high-pressure and low-temperature sensitivities of all 84 of the mutants with nutrient prototrophies.

In the present study, we first examined whether nutrient prototrophies rescued growth in the 84 deletion mutants under high pressure and low temperature in order to identify novel functional links of the genes with the regulation of nutrient permeases. Interestingly, a large proportion of the poorly characterized 12 genes had links with the uptake of nutrients under high pressure. Strikingly, all of these genes localized in the vicinity of the cell polarity and morphogenesis cluster in a recently published global genetic interaction network mapping cellular functions, and they had highly similar genetic interaction profiles, thereby suggesting that they work together as a novel functional module^31^. We demonstrated that the deletion of one of the genes, *MAY24/YPR153W*, resulted in a severe growth defect in a tryptophan auxotrophic strain under high pressure conditions^32^. This suggests that May24/Ypr153w plays a role in maintaining the functionality of Tat2. In the present study, we demonstrate that *MAY24/YPR153W* encodes a small endoplasmic reticulum (ER) resident protein that physically interacts with some nutrient permeases to ensure the functionality of substrate transport under high pressure.

## Results

### Nutrient prototrophies restored the ability for high-pressure growth in 24 mutants

To obtain insights into the mechanisms involved with high-pressure adaptation, we classified the 84 genes identified previously^24^ according to whether nutrient prototrophies for histidine, leucine, uracil, and lysine (*HIS3*, *LEU2*, *URA3*, and *LYS2*, respectively) could restore growth under high pressure and/or low temperature. All of the 84 deletion mutants were transformed with four centromere-based plasmids together, i.e., pRS313 (*HIS3*), pRS315 (*LEU2*), pRS316 (*URA3*), and pRS317 (*LYS2*), except for the *caf17/iba57*∆ mutant. Caf17/Iba57 is a mitochondrial matrix protein, and the *caf17/iba57*∆ mutation is known to cause lysine auxotrophy^33^. Therefore, it was only transformed with the three plasmids carrying *HIS3*, *LEU2*, and *URA3*, and the medium was supplemented with lysine. Individual prototrophic mutants were cultured under atmospheric pressure (0.1 MPa and 25 °C), high pressure (25 MPa and 25 °C), or low temperature (0.1 MPa and 15 °C). We found that nutrient prototrophy greatly enhanced the growth of the 24 deletion mutants under high pressure by 2 to 7 times (Table 1, closed circles; *ERG2* [*R*_HP_ = 1.8] and *PAR32* [*R*_HP_ = 1.9] are included for their importance) compared with the auxotrophic strain, although it was moderately effective at enhancing growth under low temperature (up to twofold, Table 1, open circles). Therefore, we assumed that nutrient uptake was severely damaged by high pressure in these mutants as the limiting factor for growth, thereby suggesting that the 24 genes are required for the integrity of nutrient permeases under high pressure. In particular, among the 12 poorly characterized genes responsible for high-pressure growth^24^, eight genes were ranked within the top 24 in the list for nutrient prototrophies that conferred growth, i.e., *MTC4/YBR255W*, *YDL172C*, *PAR32/YDL173W*, *MTC6/YHR151C*, *MTC2/YKL098W*, *YLR087C/CSF1*, *DLT1/YMR126C*, and *MAY24*/*YPR153W* (Table 1)*. YDL172C* and *PAR32/YDL173W* were mutually overlapping on the opposite DNA strand, so this was a single deletion mutant. Therefore, we found an unexpected link between seven poorly characterized genes and nutrient availability.

**Table 1:** Growth profiles of the deletion mutants with nutrient auxotrophies or prototorophies under high pressure and low temperature.

Name		OD_600_ at 24 h, auxotroph			OD_600_ at 24 h, prototroph			Growth enhancement by prototrophies				
Standard	Systematic	0.1 MPa, 25 °C	25 MPa, 25 °C	0.1 MPa, 15 °C	0.1 MPa, 25 °C	25 MPa, 25 °C	0.1 MPa, 15 °C	*R*_Cont_^a^	*R*_HP_^b^		*R*_LT_^c^	
Wild-type		4.1 ± 0.2	1.6 ± 0.3	1.2 ± 0.2	3.8 ± 0.3	1.8 ± 0.3	1.0 ± 0.2	0.9 ± 0.1	1.1 ± 0.1		0.9 ± 0.2	
**Amino acid biosynthesis**												
*LEU3*	*YLR451W*	4.2 ± 0.1	0.4 ± 0.1	1.4 ± 0.1	3.5 ± 0.2	1.1 ± 0.1	0.9 ± 0.1	0.8 ± 0.1	3.2 ± 0.6	●	0.6 ± 0.1	
*THR4*	*YCR053W*	3.6 ± 0.7	0.2 ± 0.1	0.3 ± 0.3	3.2 ± 0.4	0.2 ± 0.0	0.3 ± 0.0	0.9 ± 0.2	2.2 ± 1.6	●	1.6 ± 1.1	○
*ARO2*	*YGL148W*	4.0 ± 0.1	0.2 ± 0.0	0.4 ± 0.0	2.6 ± 0.4	0.3 ± 0.1	0.3 ± 0.0	0.7 ± 0.1	1.3 ± 0.3		0.7 ± 0.1	
*SER1*	*YOR184W*	4.2 ± 0.1	0.9 ± 0.3	0.8 ± 0.2	3.8 ± 0.5	1.0 ± 0.1	0.9 ± 0.2	0.9 ± 0.1	1.2 ± 0.4		1.2 ± 0.5	○
*TRP1*	*YDR007W*	4.0 ± 0.0	0.2 ± 0.1	0.4 ± 0.0	3.4 ± 0.2	0.2 ± 0.0	0.3 ± 0.0	0.8 ± 0.1	1.1 ± 0.4		0.7 ± 0.1	
*ARO1*	*YDR127W*	3.8 ± 0.2	0.1 ± 0.0	0.5 ± 0.0	2.4 ± 0.2	0.2 ± 0.0	0.3 ± 0.1	0.6 ± 0.0	1.1 ± 0.1		0.8 ± 0.2	
*TRP4*	*YDR354W*	4.4 ± 0.1	0.4 ± 0.1	0.9 ± 0.4	4.0 ± 0.3	0.3 ± 0.1	0.8 ± 0.3	0.9 ± 0.1	0.7 ± 0.2		0.9 ± 0.1	
*TRP5*	*YGL026C*	4.3 ± 0.2	0.4 ± 0.1	0.5 ± 0.2	4.0 ± 0.2	0.3 ± 0.0	0.5 ± 0.2	0.9 ± 0.1	0.7 ± 0.1		1.0 ± 0.2	
*TRP2*	*YER090W*	4.3 ± 0.3	0.4 ± 0.0	1.1 ± 0.1	3.5 ± 0.4	0.3 ± 0.1	0.7 ± 0.2	0.8 ± 0.1	0.7 ± 0.1		0.7 ± 0.3	
*HOM3*	*YER052C*	3.9 ± 0.7	0.7 ± 0.0	0.8 ± 0.2	3.8 ± 0.5	0.4 ± 0.1	0.6 ± 0.1	1.0 ± 0.1	0.6 ± 0.2		0.8 ± 0.3	
**TORC1 signaling**												
*GTR1*	*YML121W*	4.3 ± 0.2	0.2 ± 0.0	0.6 ± 0.3	3.1 ± 0.3	0.2 ± 0.0	0.5 ± 0.4	0.7 ± 0.1	0.9 ± 0.3		0.9 ± 0.3	
*GTR2*	*YGR163W*	4.4 ± 0.2	0.2 ± 0.0	0.6 ± 0.3	3.0 ± 0.5	0.2 ± 0.1	0.6 ± 0.5	0.7 ± 0.1	0.8 ± 0.4		0.8 ± 0.4	
*EGO1*	*YKR007W*	4.2 ± 0.3	0.3 ± 0.1	0.6 ± 0.4	3.3 ± 0.2	0.2 ± 0.0	0.6 ± 0.4	0.8 ± 0.0	1.0 ± 0.2		1.0 ± 0.1	
*EGO3*	*YBR077C*	3.7 ± 0.2	0.6 ± 0.1	0.9 ± 0.2	3.1 ± 0.2	0.4 ± 0.0	0.7 ± 0.4	0.9 ± 0.0	0.7 ± 0.2		0.8 ± 0.3	
**Mitochondrial function**												
*MRF1*	*YGL143C*	3.9 ± 0.5	0.3 ± 0.1	0.8 ± 0.4	3.7 ± 0.3	1.1 ± 0.1	1.1 ± 0.1	1.0 ± 0.1	3.8 ± 1.6	●	1.8 ± 1.2	○
*MRPL38*	*YKL170W*	3.5 ± 0.3	0.5 ± 0.1	0.6 ± 0.1	3.4 ± 0.1	1.0 ± 0.1	0.6 ± 0.0	1.0 ± 0.1	2.0 ± 0.6	●	1.0 ± 0.3	
*CAF17*	*YJR122W*	3.6 ± 0.2	0.3 ± 0.0	0.6 ± 0.1	2.7 ± 0.3	0.4 ± 0.1	0.6 ± 0.1	0.8 ± 0.1	1.5 ± 0.1		1.0 ± 0.0	
*ATP15*	*YPL271W*	3.2 ± 0.2	0.5 ± 0.1	0.7 ± 0.1	3.2 ± 0.1	0.8 ± 0.0	0.7 ± 0.1	1.0 ± 0.1	1.5 ± 0.3		1.1 ± 0.3	
*MDJ1*	*YFL016C*	3.4 ± 0.2	0.5 ± 0.0	0.6 ± 0.1	2.1 ± 0.4	0.6 ± 0.1	0.5 ± 0.1	0.6 ± 0.1	1.1 ± 0.2		0.8 ± 0.2	
*MRPL22*	*YNL177C*	4.1 ± 0.2	1.6 ± 0.1	1.6 ± 0.1	3.9 ± 0.3	1.7 ± 0.0	1.1 ± 0.1	0.9 ± 0.1	1.1 ± 0.1		0.7 ± 0.1	
*MRP51*	*YPL118W*	3.9 ± 0.0	0.9 ± 0.1	0.7 ± 0.1	3.2 ± 0.3	0.9 ± 0.1	0.7 ± 0.1	0.8 ± 0.1	1.0 ± 0.2		1.1 ± 0.0	
*ACO1*	*YLR304C*	3.5 ± 0.0	0.4 ± 0.1	0.9 ± 0.0	2.1 ± 0.0	0.4 ± 0.0	0.4 ± 0.0	0.6 ± 0.0	1.0 ± 0.3		0.4 ± 0.0	
*MSY1*	*YPL097W*	4.0 ± 0.0	1.4 ± 0.1	1.1 ± 0.0	3.6 ± 0.0	1.2 ± 0.1	1.0 ± 0.1	0.9 ± 0.0	0.9 ± 0.1		0.9 ± 0.1	
**Actin organization/bud formation**												
*LTE1*	*YAL024C*	4.2 ± 0.1	0.6 ± 0.1	1.1 ± 0.2	4.1 ± 0.4	0.7 ± 0.3	0.8 ± 0.4	1.0 ± 0.1	1.1 ± 0.3		0.7 ± 0.2	
*HOF1*	*YMR032W*	3.9 ± 0.4	0.7 ± 0.2	1.5 ± 0.3	3.9 ± 0.3	0.8 ± 0.2	1.0 ± 0.3	1.0 ± 0.0	1.2 ± 0.1		0.7 ± 0.1	
*CLA4*	*YNL298W*	3.7 ± 0.1	1.0 ± 0.3	0.8 ± 0.0	3.5 ± 0.2	1.2 ± 0.7	0.6 ± 0.1	1.0 ± 0.1	1.2 ± 0.3		0.8 ± 0.2	
*CDC50*	*YCR094W*	4.4 ± 0.1	1.4 ± 0.2	1.4 ± 0.2	4.2 ± 0.3	1.6 ± 0.1	1.1 ± 0.1	0.9 ± 0.0	1.2 ± 0.2		0.8 ± 0.2	
*SLM6*	*YBR266C*	4.1 ± 0.1	1.1 ± 0.3	0.8 ± 0.2	3.7 ± 0.5	1.5 ± 0.3	0.8 ± 0.2	0.9 ± 0.1	1.3 ± 0.2		0.9 ± 0.1	
**Membrane trafficking**												
*VID24*	*YBR105C*	3.5 ± 0.3	0.3 ± 0.0	1.0 ± 0.0	3.3 ± 0.2	1.4 ± 0.1	1.0 ± 0.0	1.0 ± 0.0	4.6 ± 0.4	●	1.0 ± 0.1	
*ERG3*	*YLR056W*	3.8 ± 0.1	0.4 ± 0.0	0.6 ± 0.2	4.1 ± 0.2	1.6 ± 0.1	0.9 ± 0.1	1.1 ± 0.1	3.6 ± 0.2	●	1.4 ± 0.3	○
*ERG24*	*YNL280C*	3.7 ± 0.5	0.6 ± 0.1	0.5 ± 0.0	3.9 ± 0.6	1.4 ± 0.2	0.8 ± 0.2	1.1 ± 0.1	2.4 ± 0.5	●	1.5 ± 0.4	○
*ERG6*	*YML008C*	3.6 ± 0.5	0.9 ± 0.3	0.6 ± 0.2	3.3 ± 0.3	1.7 ± 0.3	0.8 ± 0.0	0.9 ± 0.1	2.2 ± 0.7	●	1.5 ± 0.5	○
*CHC1*	*YGL206C*	1.4 ± 0.1	0.2 ± 0.0	0.2 ± 0.0	1.6 ± 0.1	0.3 ± 0.1	0.4 ± 0.0	1.2 ± 0.2	2.0 ± 1.0	●	1.7 ± 0.4	○
*ERG2*	*YMR202W*	2.9 ± 0.7	0.5 ± 0.1	0.3 ± 0.0	3.6 ± 0.5	0.9 ± 0.4	0.5 ± 0.1	1.3 ± 0.4	1.8 ± 0.5	●	1.5 ± 0.3	○
*ERG5*	*YMR015C*	3.8 ± 0.0	0.5 ± 0.1	0.6 ± 0.3	3.5 ± 0.0	0.8 ± 0.1	0.8 ± 0.1	0.9 ± 0.0	1.7 ± 0.2		1.8 ± 1.4	○
*SAC1*	*YKL212W*	3.0 ± 0.2	0.4 ± 0.0	0.3 ± 0.1	2.5 ± 0.0	0.5 ± 0.3	0.3 ± 0.1	0.8 ± 0.1	1.3 ± 0.7		0.9 ± 0.1	
*VPS45*	*YGL095C*	4.0 ± 0.3	1.3 ± 0.2	1.0 ± 0.2	3.7 ± 0.2	1.7 ± 0.2	0.8	0.9 ± 0.0	1.3 ± 0.2		0.7 ± 0.2	
*VPS54*	*YDR027C*	2.0 ± 0.3	0.3 ± 0.1	0.4 ± 0.1	2.2 ± 0.1	0.3 ± 0.0	0.5 ± 0.0	1.1 ± 0.2	1.1 ± 0.3		1.1 ± 0.2	
*SEC22*	*YLR268W*	3.1 ± 0.1	0.3 ± 0.1	0.6 ± 0.2	2.1 ± 0.2	0.3 ± 0.0	0.3 ± 0.1	0.7 ± 0.1	0.9 ± 0.3		0.6 ± 0.1	
*AKR1*	*YDR264C*	3.4 ± 0.3	0.4 ± 0.1	0.5 ± 0.1	2.9 ± 0.4	0.3 ± 0.1	0.3 ± 0.1	0.8 ± 0.1	0.7 ± 0.2		0.6 ± 0.1	
*PEP5*	*YMR231W*	2.6 ± 0.2	0.4 ± 0.1	0.6 ± 0.0	1.9 ± 0.2	0.2 ± 0.0	0.5 ± 0.1	0.8 ± 0.1	0.7 ± 0.2		0.8 ± 0.1	
*PEP3*	*YLR148W*	2.7 ± 0.2	0.4 ± 0.1	0.6 ± 0.1	2.1 ± 0.3	0.2 ± 0.1	0.4 ± 0.1	0.8 ± 0.1	0.6 ± 0.2		0.6 ± 0.1	
*VPS34*	*YLR240W*	2.3 ± 0.2	0.2 ± 0.0	0.4 ± 0.2	1.1 ± 0.6	0.1 ± 0.0	0.2 ± 0.1	0.5 ± 0.3	0.5 ± 0.3		0.5 ± 0.1	
**Inositol phosphate metabolism**												
*ARG82*	*YDR173C*	2.9 ± 0.8	0.2 ± 0.2	0.2 ± 0.2	1.9 ± 0.4	0.3 ± 0.1	0.3 ± 0.1	0.7 ± 0.2	2.1 ± 1.4	●	1.4 ± 0.8	○
*KCS1*	*YDR017C*	3.1 ± 0.1	0.6 ± 0.2	0.6 ± 0.2	3.6 ± 0.3	1.0 ± 0.2	0.4 ± 0.1	1.1 ± 0.1	1.6 ± 0.3		0.8 ± 0.0	
*PHO88*	*YBR106W*	3.8 ± 0.1	1.3 ± 0.1	0.9 ± 0.2	3.6 ± 0.2	1.6 ± 0.1	0.8 ± 0.2	0.9 ± 0.1	1.2 ± 0.1		0.9 ± 0.1	
*PLC1*	*YPL268W*	3.6 ± 0.2	0.3 ± 0.1	0.4 ± 0.1	2.2 ± 1.0	0.3 ± 0.2	0.3 ± 0.2	0.6 ± 0.3	0.9 ± 0.4		0.9 ± 0.3	
**Transcriptio/mRNA degradation**												
*SHE3*	*YBR130C*	3.5 ± 0.1	0.4 ± 0.0	0.4 ± 0.0	2.3 ± 0.2	1.0 ± 0.2	0.4 ± 0.1	0.7 ± 0.1	2.3 ± 0.4	●	1.2 ± 0.2	○
*SAP155*	*YFR040W*	4.0 ± 0.2	0.5 ± 0.1	0.6 ± 0.1	3.6 ± 0.0	1.1 ± 0.2	0.8 ± 0.0	0.9 ± 0.0	2.2 ± 0.7	●	1.3 ± 0.2	○
*SLM3*	*YDL033C*	4.3 ± 0.1	0.4 ± 0.2	0.9 ± 0.2	3.9 ± 0.5	0.8 ± 0.1	1.0 ± 0.1	0.9 ± 0.1	2.2 ± 0.9	●	1.1 ± 0.1	
*TAF14*	*YPL129W*	1.8 ± 0.3	0.3 ± 0.0	0.3 ± 0.1	2.4 ± 0.2	0.5 ± 0.1	0.5 ± 0.1	1.3 ± 0.1	1.8 ± 0.3	●	1.4 ± 0.2	○
*SRB5*	*YGR104C*	3.0 ± 0.4	0.3 ± 0.1	0.6 ± 0.3	2.5 ± 0.2	0.4 ± 0.0	0.5 ± 0.0	0.8 ± 0.1	1.3 ± 0.4		1.1 ± 0.5	
*SNF6*	*YHL025W*	3.6 ± 0.1	0.5 ± 0.1	0.8 ± 0.1	3.0 ± 0.3	0.6 ± 0.0	0.7	0.8 ± 0.1	1.3 ± 0.2		0.9 ± 0.2	
*POP2*	*YNR052C*	2.8 ± 0.1	0.3 ± 0.0	0.6 ± 0.1	2.5 ± 0.1	0.4 ± 0.1	0.5 ± 0.1	0.9 ± 0.0	1.2 ± 0.3		0.9 ± 0.0	
*ELF1*	*YKL160W*	4.1 ± 0.1	0.3 ± 0.0	0.8 ± 0.1	3.1 ± 0.1	0.3 ± 0.0	0.5 ± 0.1	0.8 ± 0.0	1.1 ± 0.1		0.6 ± 0.0	
*SNF1*	*YDR477W*	3.9 ± 0.1	0.4 ± 0.1	1.2 ± 0.3	3.1 ± 0.7	0.3 ± 0.2	0.5 ± 0.3	0.8 ± 0.2	0.8 ± 0.6		0.5 ± 0.4	
*CCR4*	*YAL021C*	3.4 ± 0.2	0.6 ± 0.0	0.7 ± 0.1	3.0 ± 0.2	0.5 ± 0.0	0.5 ± 0.1	0.9 ± 0.1	0.8 ± 0.1		0.8 ± 0.2	
*RPB4*	*YJL140W*	2.8 ± 0.1	0.3 ± 0.0	0.7 ± 0.1	1.6 ± 0.2	0.2 ± 0.1	0.4 ± 0.1	0.6 ± 0.1	0.8 ± 0.1		0.5 ± 0.2	
*CDC73*	*YLR418C*	3.8 ± 0.2	0.4 ± 0.0	0.9 ± 0.1	2.5 ± 0.4	0.3 ± 0.1	0.7 ± 0.2	0.6 ± 0.1	0.7 ± 0.2		0.7 ± 0.2	
*PAF1*	*YBR279W*	2.2 ± 0.1	0.3 ± 0.0	0.4 ± 0.0	1.8 ± 0.2	0.2 ± 0.1	0.3 ± 0.0	0.8 ± 0.1	0.7 ± 0.2		0.9 ± 0.0	
*HFI1*	*YPL254W*	2.5 ± 0.2	0.5 ± 0.1	0.6 ± 0.1	1.3 ± 0.6	0.3 ± 0.2	0.3 ± 0.2	0.5 ± 0.2	0.6 ± 0.2		0.5 ± 0.3	
*MOT2*	*YER068W*	2.9 ± 0.2	0.3 ± 0.0	0.6 ± 0.1	1.0 ± 0.1	0.1 ± 0.0	0.4 ± 0.0	0.3 ± 0.0	0.4 ± 0.1		0.7 ± 0.1	
**Ribosome**												
*RPL1B*	*YGL135W*	3.4 ± 0.1	0.4 ± 0.1	0.7 ± 0.1	2.9 ± 0.2	0.7 ± 0.3	0.5 ± 0.1	0.9 ± 0.1	1.6 ± 0.4		0.8 ± 0.0	
*RPL21A*	*YBR191W*	3.7 ± 0.1	0.6 ± 0.1	0.6 ± 0.1	2.3 ± 0.3	0.8 ± 0.1	0.5 ± 0.1	0.6 ± 0.1	1.2 ± 0.2		0.8 ± 0.1	
*RPS30B*	*YOR182C*	3.6 ± 0.1	1.5 ± 0.1	0.5 ± 0.1	2.9 ± 0.2	1.5 ± 0.2	0.5 ± 0.1	0.8 ± 0.1	1.0 ± 0.2		1.1 ± 0.4	
**Chromatin maintenance**												
*NBP2*	*YDR162C*	3.3 ± 0.4	0.2 ± 0.1	0.5 ± 0.1	2.2 ± 0.2	0.2 ± 0.0	0.4 ± 0.1	0.7 ± 0.1	0.9 ± 0.2		0.7 ± 0.1	
*YAF9*	*YNL107W*	3.7 ± 0.1	0.4 ± 0.1	0.8 ± 0.1	3.5 ± 0.1	0.5 ± 0.0	0.6 ± 0.1	0.9 ± 0.0	1.4 ± 0.3		0.8 ± 0.2	
*IES2*	*YNL215W*	4.1 ± 0.3	1.0 ± 0.1	0.9 ± 0.0	3.3 ± 0.3	0.8 ± 0.1	0.6 ± 0.1	0.8 ± 0.1	0.8 ± 0.0		0.7 ± 0.0	
*CGI121*	*YML036W*	3.2 ± 0.2	0.5 ± 0.1	0.5 ± 0.1	2.7 ± 0.2	0.8 ± 0.1	0.5 ± 0.0	0.8 ± 0.0	1.7 ± 0.4		0.9 ± 0.0	
*ARD1*	*YHR013C*	3.7 ± 0.2	0.8 ± 0.1	0.9 ± 0.2	3.6 ± 0.1	1.3 ± 0.1	0.8 ± 0.1	1.0 ± 0.1	1.6 ± 0.2		0.9 ± 0.1	
**Stress response**												
*HSP31*	*YDR533C*	3.3 ± 0.2	0.6 ± 0.1	1.0 ± 0.1	3.2 ± 0.2	1.3 ± 0.0	1.0 ± 0.1	1.0 ± 0.0	2.2 ± 0.1	●	1.0 ± 0.0	
*YDJ1*	*YNL064C*	2.4 ± 0.2	0.4 ± 0.0	0.3 ± 0.0	2.6 ± 0.2	0.5 ± 0.0	0.3 ± 0.0	1.1 ± 0.0	1.3 ± 0.1		0.9 ± 0.1	
**Poorly characterized genes**												
*MAY24*	*YPR153W*	3.5 ± 0.1	0.2 ± 0.1	0.7 ± 0.0	3.3 ± 0.1	1.4 ± 0.2	0.7 ± 0.2	1.0 ± 0.0	7.0 ± 1.7	●	1.0 ± 0.3	
*MTC4*	*YBR255W*	3.5 ± 0.1	0.3 ± 0.1	0.8 ± 0.0	3.3 ± 0.4	1.4 ± 0.1	1.0 ± 0.1	0.9 ± 0.1	5.4 ± 1.1	●	1.2 ± 0.1	○
*DLT1*	*YMR126C*	3.6 ± 0.3	0.3 ± 0.1	0.7 ± 0.1	3.4 ± 0.0	1.4 ± 0.1	1.0 ± 0.1	1.0 ± 0.1	5.4 ± 1.1	●	1.4 ± 0.4	○
*MTC6*	*YHR151C*	3.6 ± 0.2	0.3 ± 0.1	0.8 ± 0.1	3.4 ± 0.2	1.2 ± 0.1	1.0 ± 0.1	0.9 ± 0.0	4.5 ± 0.6	●	1.3 ± 0.1	○
*MTC2*	*YKL098W*	3.5 ± 0.1	0.3 ± 0.1	0.9 ± 0.1	3.2 ± 0.3	1.2 ± 0.1	1.0 ± 0.1	0.9 ± 0.1	4.1 ± 0.9	●	1.2 ± 0.1	○
*CSF1*	*YLR087C*	2.4 ± 0.1	0.3 ± 0.1	0.2 ± 0.1	2.3 ± 0.2	0.6 ± 0.2	0.3 ± 0.1	1.0 ± 0.0	2.6 ± 1.5	●	1.2 ± 0.5	○
—	*YDL172C*	4.3 ± 0.5	0.5 ± 0.1	0.9 ± 0.1	3.5 ± 0.1	1.1 ± 0.3	0.8 ± 0.1	0.8 ± 0.1	2.3 ± 0.4	●	0.9 ± 0.1	
*PAR32*	*YDL173W*	4.2 ± 0.3	0.6 ± 0.1	1.1 ± 0.2	3.4 ± 0.1	1.1 ± 0.2	0.8 ± 0.0	0.8 ± 0.1	1.9 ± 0.2	●	0.8 ± 0.2	
—	*YGL218W*	3.3 ± 0.2	0.3 ± 0.0	0.5 ± 0.0	2.7 ± 0.1	0.3 ± 0.0	0.4 ± 0.0	0.8 ± 0.1	1.1 ± 0.1		0.8 ± 0.1	
*AVL9*	*YLR114C*	3.8 ± 0.5	0.2 ± 0.0	0.6 ± 0.1	2.3 ± 0.6	0.2 ± 0.1	0.4 ± 0.1	0.6 ± 0.1	0.9 ± 0.1		0.7 ± 0.1	
—	*YDR442W*	2.1 ± 0.2	0.6 ± 0.0	0.3 ± 0.1	2.5 ± 0.1	0.4 ± 0.1	0.3 ± 0.1	1.2 ± 0.1	0.6 ± 0.1		1.2 ± 0.3	○
—	*YDR008C*	4.2 ± 0.0	0.4 ± 0.1	0.3 ± 0.0	3.7 ± 0.1	0.2 ± 0.0	0.3 ± 0.0	0.9 ± 0.0	0.5 ± 0.1		1.1 ± 0.2	

^a^*R*_Cont_ represents the ratio of the OD_600_ value for a prototrophic mutant to that for the corresponding auxotrophic mutant, measured at 0.1 MPa and 25 °C for 24 h (control).

^b^*R*_HP_ represents the ratio of the OD_600_ value for a prototrophic mutant to that for the corresponding auxotrophic mutant, measured at 25 MPa and 25 °C for 24 h (high pressure).

^c^*R*_LT_ represents the ratio of the OD_600_ value for a prototrophic mutant to that for the corresponding auxotrophic mutant measured at 0.1 MPa and 15 °C for 24 h (low temperature).

Closed circles represent genes ranked in the top 24 in terms of restoration of high-pressure growth in the deletion mutants.

Open circles represent genes ranked in the top 19 in terms of restoration oflow-temprature growth in the deletion mutants.

**Figure 1:**
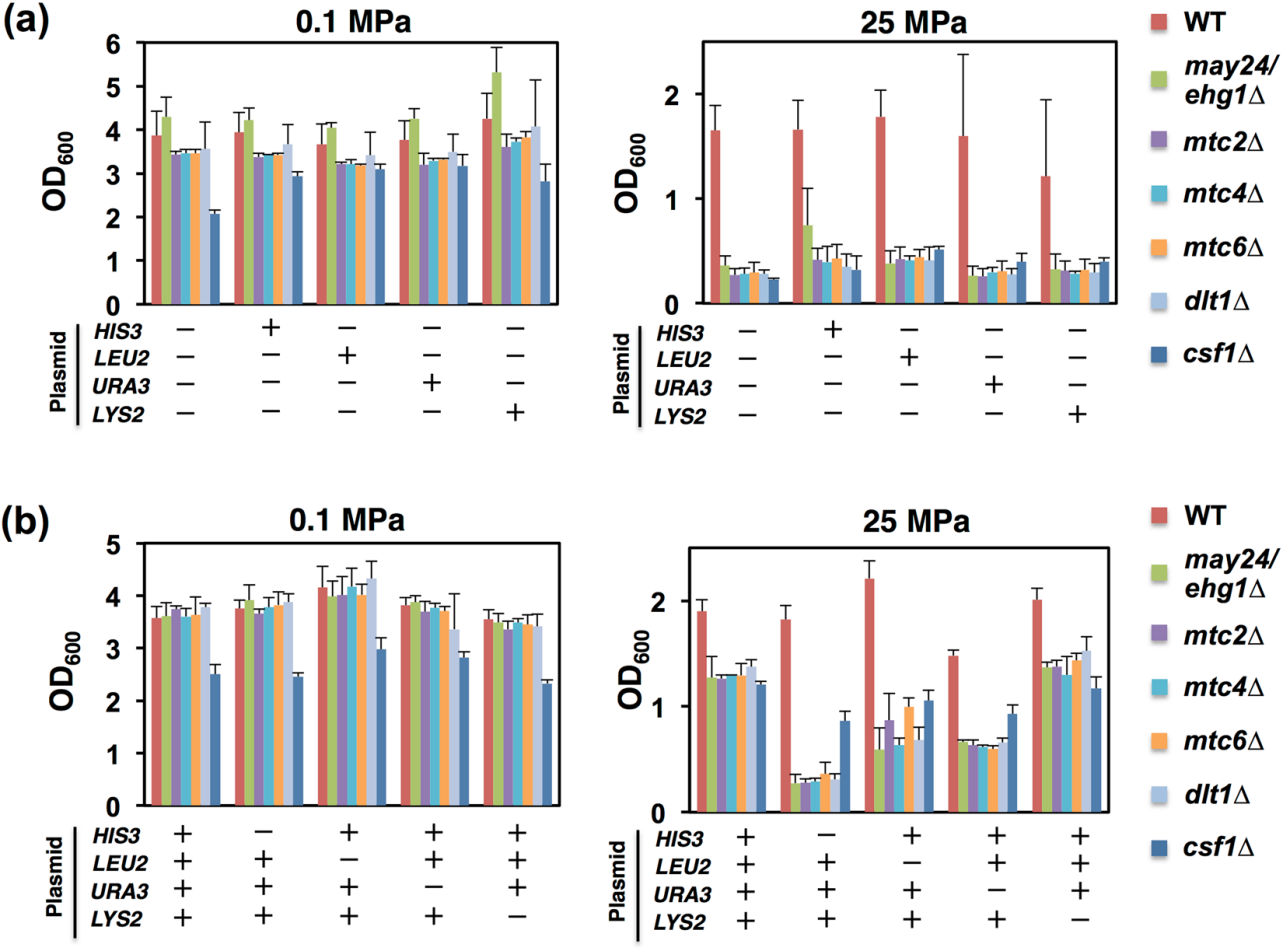
Restoration of the high-pressure growth ability of closely related mutants by conferring nutrient prototrophies. The wild-type strain and the deletion mutants with or without a single (**a**) or multiple (**b**) plasmid-borne nutrient prototrophies (*HIS3*, *LEU2*, *URA3*, and *LYS2*) were cultured at 0.1 MPa or 25 MPa and 25 °C for 24 h, starting at the OD_600_ value of 0.1. “+” or “−” indicates the presence or absence of the nutrient auxotrophic genes, respectively. Data are represented as means and standard deviations of three independent experiments.

Conferring the four nutrient prototrophies together enabled the *may24∆, mtc2∆*, *mtc4∆*, *mtc6∆*, *dlt1∆*, and *csf1∆* mutants to grow at 25 MPa almost comparably (Table 1). To analyze the minimum requirement in terms of nutrient prototrophies for high-pressure growth, the six mutants were transformed with one or three of the four plasmids carrying *HIS3*, *LEU2*, *URA3*, and *LYS2*. Low-temperature growth was not examined in our further analyses because the effect was only moderate (Table 1). We found that each one of the four plasmids introduction alone did not confer high-pressure growth ability on the mutants (Fig. 1a). However, the combined introduction of *HIS3*, *LEU2*, and *URA3* was sufficient to enable the mutants to grow at 25 MPa, whereas *LYS2* was dispensable (Fig. 1b). By contrast, the lack of one of *HIS3*, *LEU2*, and *URA3* did not confer high-pressure growth in the mutants, except partial restoration of the growth in the *csf1*∆ mutant in the absence of *HIS3*. Interestingly, the extents to which the three plasmids allowed high-pressure growth were comparable in these mutants, thereby suggesting that the six genes work in the same pathway for promoting nutrient uptake (Fig. 1).

**Figure 2:**
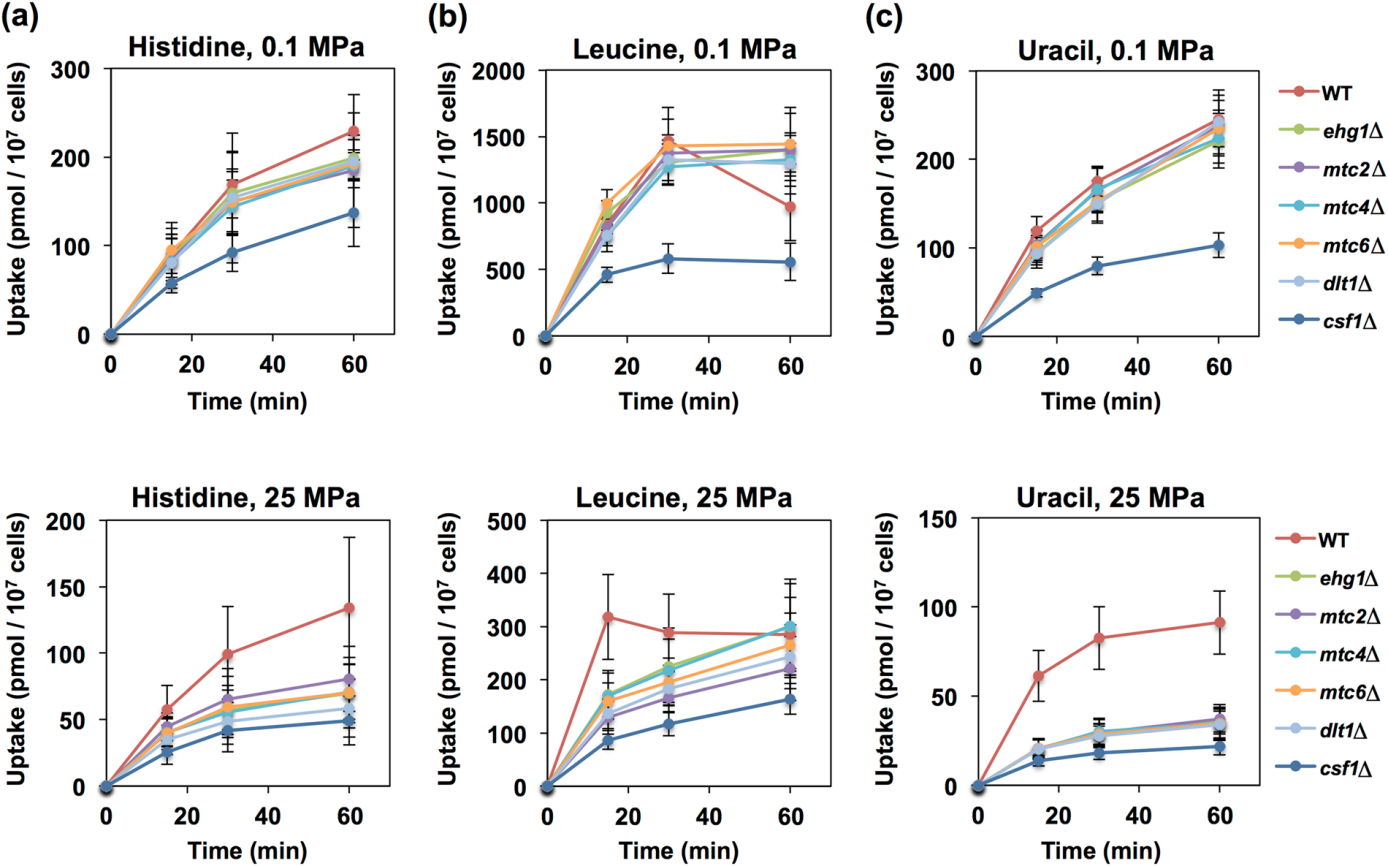
Effects of high pressure on substrate transports. The wild-type strain and deletion mutants were cultured in SC medium at 0.1 MPa or 25 MPa and 25 °C for 3 h. Following decompression, the cells were subjected to the uptake assay using [^3^H]-labeled substrates in the presence of non-labeled (**a**) histidine HCl monohydrate (2 *μ*g/mL), (**b**) leucine (9 *μ*g/mL), or (**c**) uracil (2 *μ*g/mL). Data are represented as the means and standard deviations of incorporated substrates (pmol/10^7^ cells) obtained from three independent experiments.

To investigate the effect of high pressure on nutrient uptake, we performed substrate uptake assays using ^3^H-labeled substrates. Exponentially growing cells of the wild-type and mutant strains were incubated in SC medium for 3 h at 0.1 or 25 MPa. The uptake assay was performed at 0.1 MPa after decompression (see Materials and Methods). No measurable difference in the substrate transport rates, except significant transport defects in the *csf1*∆ mutant, was observed between the wild-type and mutant strains when cultured at 0.1 MPa (Fig. 2). Csf1 was originally identified as a protein required for fermentation at low temperatures and was subsequently observed in purified mitochondria^34,35^. The *csf1*∆ mutant exhibits a reduced growth rate even at 0.1 MPa (Fig. 1), which suggests an unknown role of Csf1 in the mitochondria toward supporting normal cell growth. Incubation of the wild-type cells at 25 MPa for 3 h attenuated the transport activities, in the following order of severity at 15-min time points (percentages indicate the relative values of substrate accumulations at 25 MPa to those at 0.1 MPa): leucine (38%) > uracil (52%) > histidine (64%). Notably, incubation at high pressure led to more profound defects in the mutants than in the wild-type strain, with substrate transport rates for leucine (18%), uracil (20%), and histidine (47%) (Fig. 2). Considering these findings with Fig. 1, we assume that the proteins encoded by the seven poorly characterized genes confer stability and/or functional robustness on nutrient permeases for efficient substrate transport under high pressure.

**Figure 3:**
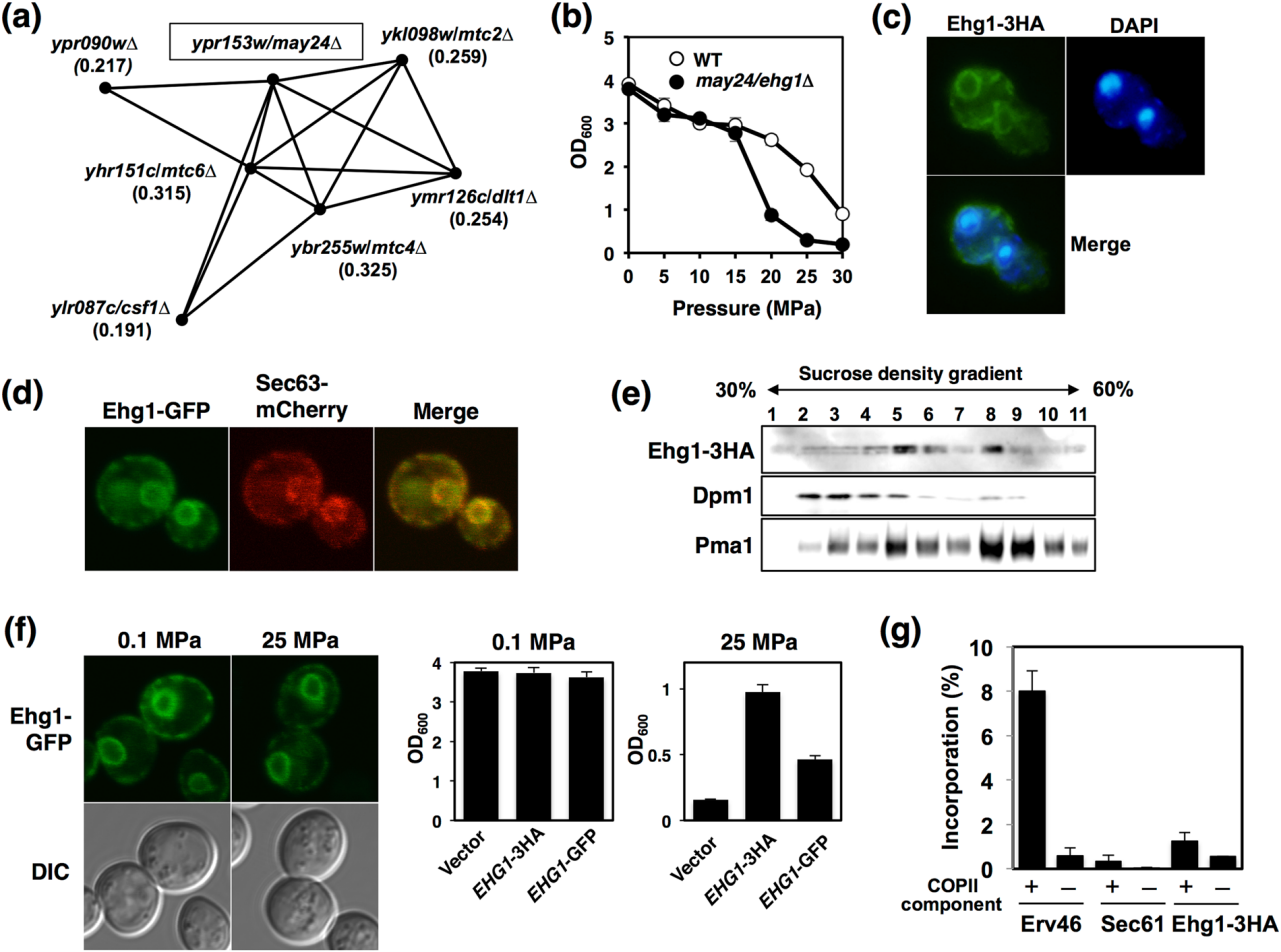
Ehg1 is a novel ER membrane protein. (**a**) Profile similarities with *MAY24/YPR153W* were calculated in TheCellMap program, and genes with the Pearson correlation coefficient (PCC) above 0.190 are represented in parentheses.^31^ (**b**) The wild-type strain and the *ehg1*∆ mutant were cultured at high pressures of up to 30 MPa and 25 °C for 24 h, and the OD_600_ values were measured. Data are represented as means and standard deviations of three independent experiments. (**c**) Immuno-staining of Ehg1-3HA using anti-HA monoclonal antibody. (**d**) Co-localization of Ehg1-GFP and an ER membrane resident protein Sec63-mCherry. (**e**) Subcellular fractions for Ehg1-3HA, Dpm1 (ER marker), and Pma1 (plasma membrane marker) from sucrose-density gradient centrifugation. (**f**) The *ehg1*∆ cells expressing Ehg1-GFP (*URA3*, *CEN*) were imaged under a confocal laser microscope after cultured at 0.1 MPa or 25 MPa and 25 °C for 24 h (left). The *ehg1*∆ cells expressing Ehg1-3HA (*URA3*, *CEN*) or Ehg1-GFP (*URA3*, *CEN*) were cultured at 0.1 MPa or 25 MPa and 25 °C for 24 h, starting at the OD_600_ value of 0.1 (middle and right). Data are represented as means and standard deviations of three independent experiments. (**g**) *In vitro* COPII budding assay on Ehg1. The ER-enriched membrane fractions prepared from the indicated strains were incubated in the presence or absence of purified COPII coat components. The incorporation of Ehg1-3HA, Erv46, and Sec61 into COPII vesicles was analyzed by immunoblotting. A percentage of each protein incorporated in the COPII vesicle fraction compared with total amount of each protein present in the reaction was plotted as a packaging efficiency. Data are represented as means and standard deviations of three independent experiments.

We found a striking coincidence with a report that demonstrated the mapping of six genes, i.e., *MTC2*, *MTC4*, *MTC6*, *DLT1*, *CSF1*, and *MAY24*, in the vicinity of the cell polarity and morphogenesis cluster according to the global network generated by elucidating the fitness of 23 million combinatorial double mutants that had highly similar genetic interaction profiles^31,36^, which suggests that their gene products work together as a novel functional module (Fig. 3a). *YPR153W* was designated as *MAY24* because of the similarity of the genetic interaction profile with the *MTC* annotated yeast genes *MTC2* and *MTC4*^31^. Previously, the deletion of *MTC* genes was shown to aggravate the mutant phenotype associated with the *cdc13-1* mutation, where the maintenance of telomere capping is defective at a restrictive temperature^37^. It was also shown that the MTC pathway genes have strong negative interactions with the aromatic amino acid biosynthesis genes *ARO1* and *ARO2*, and deletions in this pathway reduce the phenylalanine import activity and cause the mislocalization of the branched amino acid permease Bap2^31^. Our findings are consistent with those of this previous study in terms of the regulation of nutrient uptake. Because the deletion of *MAY24*/*YPR153W* led to the highest score of growth enhancement by prototrophies (*R*_HP_ = 7.0, Table 1) at 25 MPa, we decided to focus on *MAY24*/*YPR153W* in further analyses and elucidate the contribution of this protein in ER function (see below).

### May24/Ehg1/Ypr153w is an ER resident protein

May24/Ypr153w is a small protein comprising 140 amino acid residues. The auxotrophic *may24/ypr153w*∆ mutant grew at pressures of up to 15 MPa but exhibited growth defects at ≥20 MPa pressures (Fig. 3b). To determine cellular localization, we performed immuno-staining in cells expressing the *C*-terminally 3HA-tagged May24/Ypr153w, and found that it was present in the ER (Fig. 3c). In support of this, May24/Ypr153w-GFP co-localized with Sec63-mCherry, an ER marker protein (Fig. 3d). We performed biochemical assays on subcellular fractions for May24/Ypr153w-3HA, Dpm1 (ER marker), and Pma1 (plasma membrane marker) from sucrose-density gradient centrifugation and found that May24/Ypr153w overlaps with Dpm1 and partially with Pma1 (Fig. 3e). This suggests that May24/Ypr153w localizes to the ER membrane. Furthermore, some fractions in the cortical ER could be associated with the plasma membrane through uncharacterized interactions. May24/Ypr153w-GFP was partially functional and clearly remained localized in the ER membrane following the incubation of the cells at 25 MPa for 24 h (Fig. 3f). Therefore, we designated *MAY24*/*YPR153W* as *EHG* (ER-associated high-pressure growth gene) 1 for simplicity instead of the adscript description *YPR153W/MAY*2*4/EHG1*.

We speculate that Ehg1 might facilitate the accurate folding of nutrient permeases in the ER as a chaperone, thereby conferring resistance to the mechanical damage caused by high-pressure perturbation. Interestingly, *MTC*2 and *MTC6* have a genetic interaction with *SHR3*, which encodes an ER packaging chaperone that is specifically required for incorporating amino acid permeases into the coat protein complex (COP) II vesicles for transport to the cell surface^38^. Because Shr3 is not packaged into COPII-coated vesicles *in vitro*, it is considered to be a true ER resident protein, interacting only transiently with the permeases before they enter cargo vesicles^39^. Ehg1 lacks canonical ER retention motifs such as KKXX sequence at its *C*-terminal end^40^ or the arginine-based motif^41,42^. We investigated whether Ehg1 is retained in the ER by static retention mechanisms to prevent bulk flow, or it is packaged into COPII vesicles to exit from the ER to the Golgi compartments, and is retrieved by COPI retrograde vesicles into the ER. To examine the possibility of the ER exit, we performed *in vitro* COPII vesicle budding assay on Ehg1. Erv46, a protein efficiently packaged into ER-derived COPII vesicles and actively recycled from Golgi compartments to the ER in COPI vesicles^43^, and Sec61, a conserved ER protein translocation channel, were used as positive and negative controls for the budding reaction efficiency, respectively (see Materials and Methods). We found that Ehg1 was marginally incorporated into the COPII vesicles (1.3% of total Egh1-3HA proteins present in a reaction) compared with Erv46 (8% of total Erv1 proteins present in a reaction) (Fig. 3g). Ehg1 export was slightly more efficient than Sec61 export (0.3% of total Sec61 proteins used) probably because Ehg1-3HA was overexpressed in a multicopy vector in the assay. Therefore, we concluded that Ehg1 resides in the ER without export from the ER. This implies that Ehg1 may have a similar role in regulating nutrient permeases in a coordinated manner with Shr3 in the ER.

It was reported that *MTC2*, *MTC4*, *MTC6*, *DLT1*, *CSF1*, and *EHG1* deletions accumulated intracellular metabolites during *de novo* NAD^+^ biosynthesis from tryptophan, such as kynurenic acid (a branched product from kynurenine catalyzed by Bna3), 3-hydroxy-kynurenine, or 3-hydroxy-anthiranilic acid to variable degrees^31^. A previous study showed that the deletion of *BNA2* encoding tryptophan 2,3-dioxygenase in *de novo* NAD^+^ biosynthesis, or *NPT1* encoding nicotinate phosphoribosyltransferase in the salvage pathway of NAD^+^ biosynthesis, suppressed the temperature-sensitive phenotype of the *cdc13-1* mutant, suggesting that elevated NAD^+^ levels inhibit telomere capping^44^. We suspected that high concentrations of these metabolites or NAD^+^ might have adverse impacts on nutrient uptake in the *ehg1*∆ mutant under high pressure. Therefore, we examined whether deletions for *BNA2*, *BNA7* or *NPT1* suppressed the high-pressure sensitivity of the *ehg1*∆ mutant. We found that the double mutants, *ehg1*∆*bna2*∆, *ehg1*∆*bna7*∆, and *ehg1*∆*npt1*∆, normally grew at 0.1 MPa, and they still exhibited high-pressure sensitivity at 25 MPa (Fig. S1). The result suggests that the accumulation of intracellular metabolites during NAD^+^ biosynthesis is unlikely to impair nutrient uptake in the *ehg1*∆ mutant although we have not quantified the metabolite levels in the double mutants.

### Ehg1 is required for stable expression of nutrient permeases under high pressure

**Figure 4:**
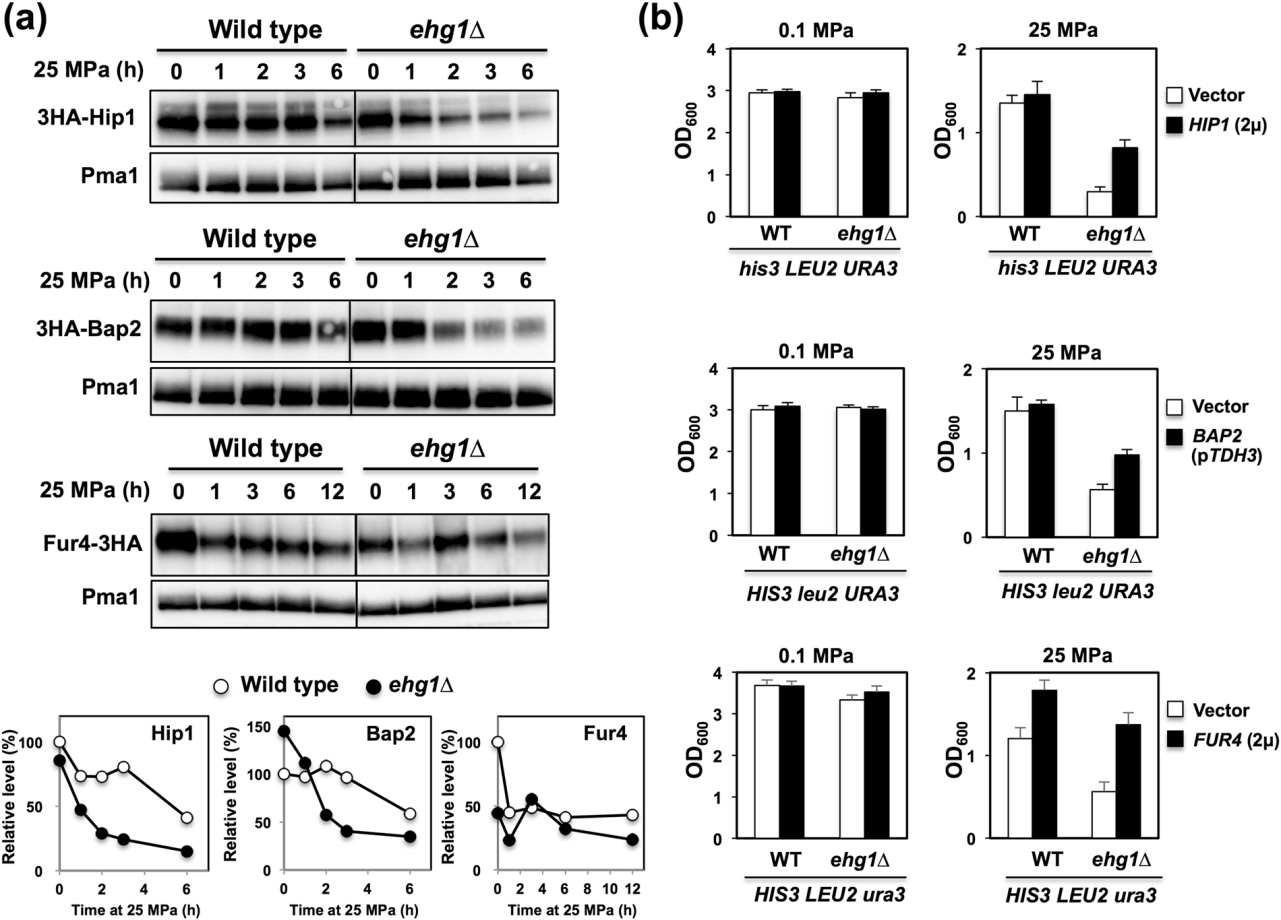
Expression of nutrient permeases and restoration of the high-pressure growth ability in the *ehg1*∆ mutant. (**a**) The wild-type strain and the *ehg1*∆ mutant expressing 3HA-Hip1, 3HA-Bap2 or Fur4-3HA were cultured at 0.1 MPa or 25 MPa, and the P13 membrane fractions were subjected to Western blot analysis. Pma1 was used as a loading control. The levels of the nutrient permeases were quantified in an ImageQuant LAS4000 mini. (**b**) The cells expressing *HIP1* or *FUR4* in a multicopy plasmid or *BAP2* driven by the *TDH3* promoter in a centromere-based plasmid were cultured at 0.1 MPa or 25 MPa for 24 h, starting at the OD_600_ value of 0.1. Data are represented as means and standard deviations of three independent experiments.

We hypothesized that Ehg1 plays a role in the expression, localization, or cell surface delivery of permeases for histidine (Hip1)^45^, leucine (Bap2 or Bap3)^46,47^, and uracil (Fur4)^48^ in high-pressure conditions. To validate this hypothesis, we analyzed the levels of Hip1, Bap2, and Fur4 during incubation at 25 MPa. The Hip1 and Bap2 levels decreased in the *ehg1*∆ mutant after pressurization, whereas they remained almost constant in the wild-type strain (Fig. 4a). Therefore, the *ehg1*∆ mutant lacks histidine and leucine under high pressure conditions. Similarly, the overexpression of either *HIP1* or *BAP2* partially restored the ability to grow under high-pressure in the *ehg1*∆ mutant in cases where two of the three nutrient synthetic genes, including *HIS3*, *LEU2* or *URA3*, were present (Fig. 4b). The results suggest that Ehg1 is required for the stable expression of Hip1 and Bap2 under high-pressure conditions. The result is consistent with our recent finding that the deletion of this gene caused a significant growth defect in a tryptophan auxotrophic strain and destabilization of Tat2 under high pressure^32^.

Fur4 levels decreased 1 h after pressurization, but remained almost constant for up to 12 h in both the wild-type and *ehg1*∆ cells (Fig. 4a). The plasma membrane Fur4 levels were comparable in the two strains during culture under high-pressure conditions. We confirmed the similar plasma membrane localization of Fur4-GFP in the wild-type strain and the *ehg1*∆ mutant under a fluorescence microscope (data not shown). We were unable to detect any considerable differences in the expression levels and distribution of Fur4 in the two strains based on repeated experiments. Nevertheless, incubation of the cells at 25 MPa for 3 h led to more a profound defect in uracil uptake in the mutant than in the wild-type strain (Fig. 2). Therefore, we propose that the lack of Ehg1 caused a subtle structural distortion, which was associated with reduced uracil transport activity at high pressure. The overexpression of *FUR4* facilitated growth at 25 MPa in both strains; however, it was more effective in the *ehg1*∆ mutant (Fig. 4b).

We considered two possibilities to account for the role of Ehg1 under high pressure. First, Ehg1 is assumed to be an ER resident protein. Therefore, it might facilitate the accurate folding of nutrient permeases in the ER as a chaperone, in turn conferring resistance to the mechanical damage caused by high-pressure perturbation. Second, Ehg1 that resides in the cortical ER might mechanically prevent nutrient permeases from pressure-induced unfolding in the plasma membrane. In yeast cells, large portions of ER called cortical ER are closely associated with plasma membrane^49^. Therefore, we examined physical interactions between Ehg1 and nutrient permeases using immunoprecipitation and the yeast two-hybrid system (see below).

### Membrane topology of Ehg1

**Figure 5:**
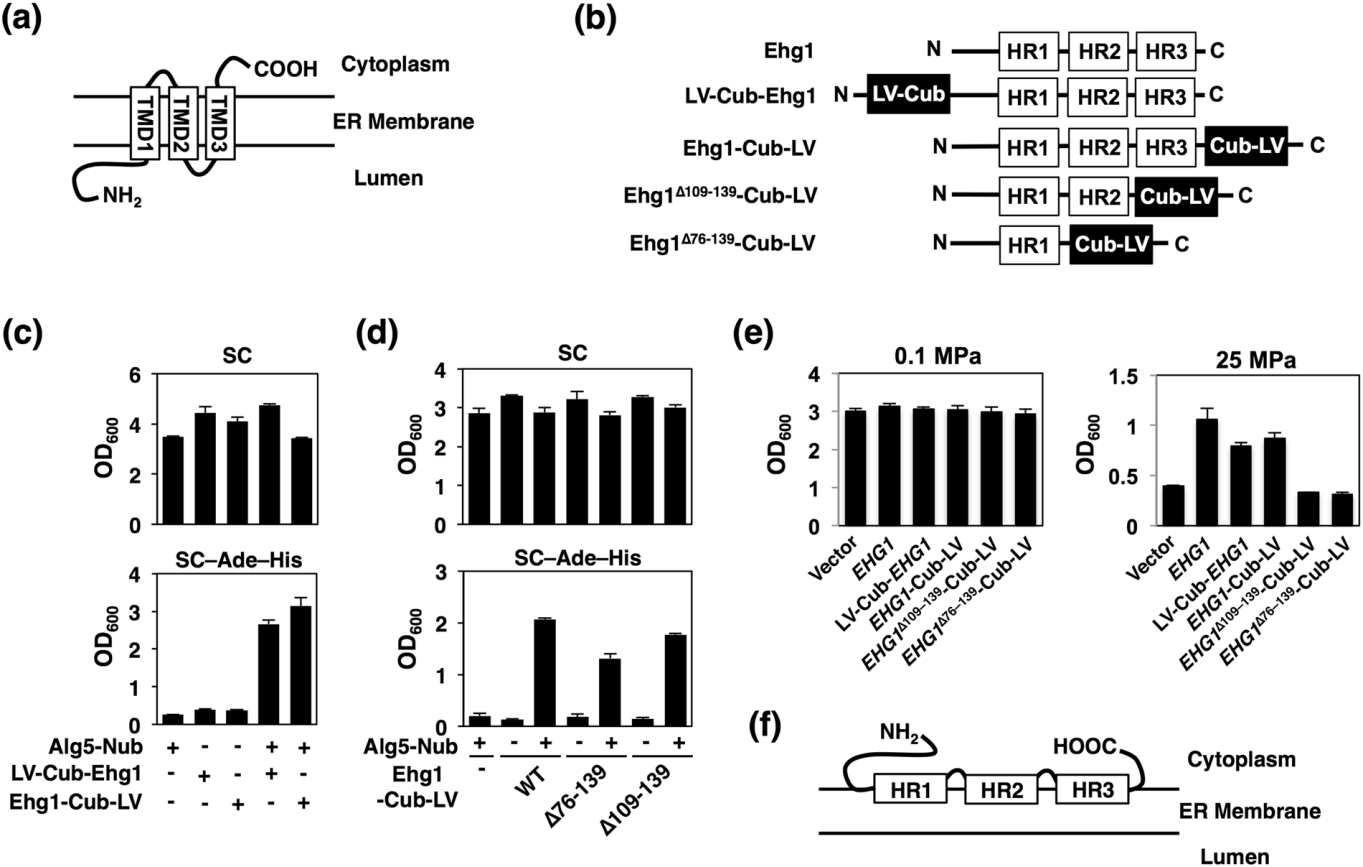
Membrane topology analysis of Ehg1. (**a**) Predicted membrane topology of Ehg1 according to TMHMM Server v. 2.0. HR, helix region. (**b**) Schematic representation of plasmid constructs for the yeast two-hybrid analysis based on the split-ubiquitin membrane system. (**c**) Strain NMY51 was transformed with the bait (Alg5-Nub) or prey [LV-Cub-Ehg1 or Ehg1-Cub-LV] plasmids and was cultured in SC medium or SC medium lacking adenine and histidine (SC–Ade–His). Data are represented as means and standard deviations of three independent experiments. (**d**) Strain NMY51 was transformed with the bait (Alg5-Nub) or the mutant forms of prey (Ehg1-Cub-LV ∆76–139 or Ehg1-Cub-LV ∆109–139) plasmids and was cultured in SC medium or SC–Ade–His medium. “+” or “−” indicates the presence or absence of the plasmid, respectively. Data are represented as means and standard deviations of three independent experiments. (**e**) The *ehg1*∆ mutant harboring plasmids used in the membrane topology analysis was cultured at 0.1 MPa or 25 MPa for 24 h, starting at the OD_600_ value of 0.1. Data are represented as means and standard deviations of three independent experiments. (**f**) Peripheral ER membrane localization of Ehg1 proposed by the yeast two-hybrid analysis.

To gain insights into the mechanistic role of Ehg1 in the ER, we next analyzed the membrane topology of Ehg1. According to programs for predicting transmembrane helices and topology of proteins, i.e., TMHMM Server v. 2.0 (http://www.cbs.dtu.dk/services/TMHMM/)^50^ and SOSUI (http://harrier.nagahama-i-bio.ac.jp/sosui/)^51^, Ehg1 was predicted to have three TMDs with a long *N*-terminal tail (residues 1–44) facing the ER lumen, a very short loop (8 amino acid residues) between TMD1 and TMD2, and a short *C*-terminal tail facing the cytoplasm (Fig. 5a). We considered whether this prediction was physically relevant. Therefore, we performed topology analysis using a yeast two-hybrid membrane protein system exploiting the split-ubiquitin mechanism (see Materials and Methods). Two plasmids expressing LexA-VP16 (LV)-Cub-Ehg1 (*N*-terminally LV-Cub-tagged Ehg1) or Ehg1-Cub-LV (*C*-terminally LV-Cub-tagged Ehg1) were constructed as bait vectors (Fig. 5b). A control plasmid expressing Alg5-Nub, which is an integral ER membrane protein^52^, was used as a prey vector. These fusion proteins were expressed in strain NMY51, and induction of the reporter genes (*ADE2* and *HIS3*) was evaluated by examining cell growth in SC medium lacking adenine and histidine (SC-Ade–His). We found no induction of the reporter genes when Alg5-Nub, LV-Cub-Ehg1, or Ehg1-Cub-LV was solely expressed in strain NMY51 (Fig. 5c). Therefore, self-activation did not occur upon the expression of individual fusion proteins alone. Importantly, the reporter genes were induced by the co-expression of LV-Cub-Ehg1 with Alg5-Nub, and they were also induced by the co-expression of Ehg1-Cub-LV with Alg5-Nub (Fig. 5c). This suggests that both the *N*- and *C*-terminal domains of Ehg1 face the cytoplasmic side, which is inconsistent with the prediction that Ehg1 might have three TMDs with a luminal *N*-terminal tail (Fig. 5a). In the following, the predicted TMDs are referred to as helix regions (HRs, i.e., HR1–3). To further elucidate the membrane topology, we constructed additional plasmids bearing genes encoding truncated forms of Ehg1, i.e., Ehg1_∆76–139_-Cub-LV lacking HR2 and HR3 and Ehg1_∆109–139_-Cub-LV lacking HR3 (Fig. 5b). Expression of these fusion proteins alone did not induce expression of the reporter gene (Fig. 5d). We found that the expression of Ehg1_∆76–139_-Cub-LV or Ehg1_∆109–139_-Cub-LV effectively induced the reporter genes when Alg5-Nub was co-expressed (Fig. 5d). Therefore, we suggest that each *C*-terminal end of Ehg1_∆76–139_ or Ehg1_∆109–139_ faces the cytoplasm. We confirmed that LV-Cub-Ehg1 and Ehg1-Cub-LV were functional in terms of their ability to restore the growth of the *ehg1*∆ mutant at 25 MPa; however, Ehg1_∆76–139_-Cub-LV and Ehg1_∆109–139_-Cub-LV were not functional (Fig. 5e). This result suggested that although the *N*-terminal domain–HR1 is sufficient for localization to the ER membrane, it is insufficient to exert any functionality for allowing cells to grow under high pressure. Based on these results, we propose a model for the topology of Ehg1 in Fig. 5f, which shows that this protein may be a peripheral membrane protein that binds the ER membrane via HR1–3.

### Phe19 and Pro17/Pro20 of Ehg1 are critical for cell growth under high pressure

**Figure 6:**
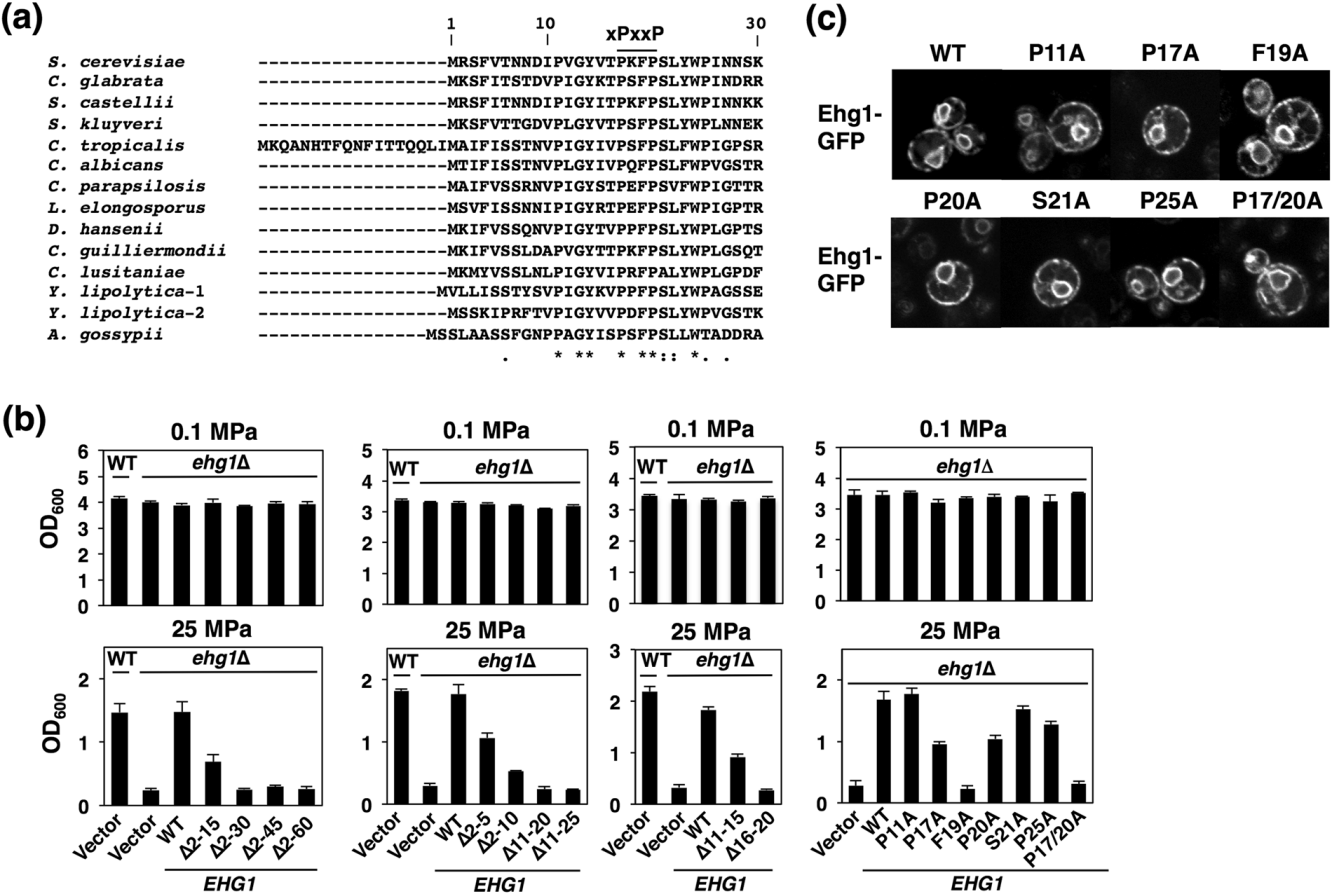
Phe19 in the *N*-terminal domain is crucially important for Ehg1 to allow high-pressure growth. (**a**) Alignment of Ehg1 homologs across various yeast species. (**b**) Effects of truncations or point mutations of Ehg1 during high-pressure growth. The *ehg1*∆ mutant expressing the indicated plasmid carrying the mutation was cultured at 0.1 MPa or 25 MPa and 25 °C for 24 h, starting at the OD_600_ value of 0.1. Data are represented as means and standard deviations of three independent experiments. (**c**) Subcellular localization of the mutant forms of Ehg1-GFP observed under a confocal laser microscope.

Ehg1 has a minimum Src homology 3 domain-binding consensus sequence (X-Pro-X-X-Pro^53,54^, in the putative *N*-terminal cytoplasmic tail. However, this interaction is questionable because the first and fourth residues are not aliphatic amino acids (Fig. 6a). Interestingly, this motif is highly conserved among Ehg1 homologues in species related to *S. cerevisiae* (Fig. 6a), which suggests that Ehg1 has a common role in yeast physiology. Among them, 30 *Debaryomyces hansenii* strains and 40 *Candida* strains have been isolated from sediment samples collected at deep-sea floors around the northwest Pacific Ocean^55^. To examine the role of the *N*-terminal domain, we constructed a series of deletion mutants for Ehg1 Δ2–15, Δ2–30, Δ2–45, and Δ2–60, and their growth was examined at 25 MPa (Fig. 6b). The wild-type cells (*ehg1*∆ mutant harboring the *EHG1* plasmid) grew efficiently at 25 MPa, but the Ehg1Δ2–15 cells had reduced growth rates, whereas the Ehg1Δ2–30, Δ2–45, and Δ2–60 cells no longer grew at 25 MPa, thereby suggesting that amino acid residues 2–30 are indispensable. Next, we created additional deletion mutants, i.e., Δ2–5, Δ2–10, Δ11–20, Δ11–25, Δ11–15, and Δ16–20, to identify the critical amino acid residues between P11 and P25 (Fig. 6b). We then substituted alanine for the amino acid residues within this region of Ehg1. Consequently, a single F19A change appeared to cause a dramatic growth defect under high pressure and a combined P17A/P20A change caused a similar defect (Fig. 6b). These point mutations did not change the ER localization of Ehg1-GFP (Fig. 6c). These results suggest that Ehg1 interacts with nutrient permeases and/or binding partners via F19 and/or P17/P20 to positively affect the integrity of the permeases and possibly any other amino acid permeases under high pressure.

### Physical interactions between Ehg1 and nutrient permeases

**Figure 7:**
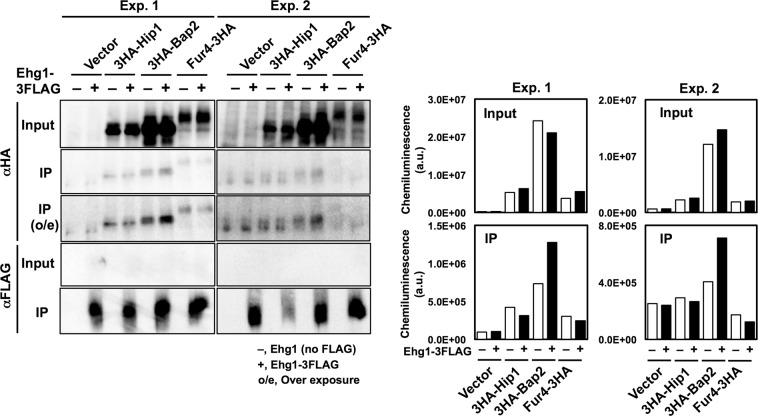
Co-immunoprecipitation of Ehg1 and nutrient permeases. The S13 fractions from the *ehg1*∆ cells expressing Ehg1-3FLAG and one among 3HA-Hip1, 3HA-Bap2, or Fur4-3HA were subjected to immunoprecipitation using anti-FLAG M2 magnetic beads. IP, immunoprecipitates; o/e, over exposure (left). The full-length images are shown in Figs. S8 and S9. The signal intensities (arbitrary units) of the nutrient permeases were quantified in an ImageQuant LAS4000 mini with defined parameter settings for data collection (right). Data of two independent experiments (Exp.1 and 2) are shown.

To investigate whether Ehg1 physically interacts with nutrient permeases, we performed co-immunoprecipitation of Ehg1 with Hip1, Bap2, and Fur4. The S13 fractions (removal of the plasma membrane) were obtained from *ehg1*∆ cells co-expressing Ehg1–3FLAG and each one of 3HA-Hip1, 3HA-Bap2, or Fur4–3HA. Ehg1-3FLAG was collected using anti-FLAG M2 magnetic beads following the solubilization of the membrane with 1% Triton X-100. Although we failed to observe substantial amounts of the permeases in the Ehg1 immunoprecipitates, a trace amount of Bap2 was detected in the chemiluminescence measurement (Fig. 7). Hip and Fur4 were not detected. The result suggested that the interactions between Ehg1 and the nutrient permeases could be weak or highly transient.

**Figure 8:**
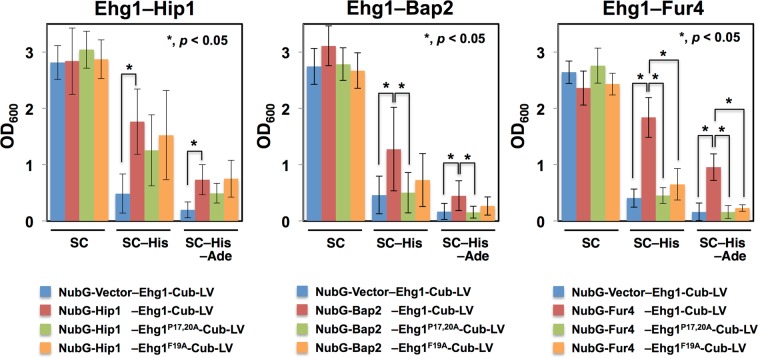
Physical interactions between Ehg1 and nutrient permeases. The yeast two-hybrid analysis was based on the split-ubiquitin mechanism. Strain NMY51 was co-transformed with the bait and prey plasmids. The wild-type Ehg1-Cub-LV, and its variants P17/20 A or F19A were used as the preys, and NubG-Hip1, NubG-Bap2, and NubG-Fur4 were used as the baits. The transformant cells were cultured in SC medium with or without histidine and adenine for 24 h starting at the OD_600_ value of 0.1. Data are represented as means and standard deviations of three independent experiments. Student’s *t*-test was used for statistical analyses.

Therefore, we explored another approach to investigate *in vivo* physical interactions between Ehg1 and nutrient permeases using the yeast two-hybrid membrane protein system based on the split-ubiquitin mechanism. Plasmids expressing LV-Cub-Ehg1 or Ehg1-Cub-LV were used as bait vectors, and plasmids expressing NubG-Bap2, NubG-Hip1, or NubG-Fur4 were used as prey vectors. The fusion proteins were expressed in strain NMY51, and induction of the reporter genes (*ADE2* and *HIS3*) was evaluated by examining cell growth in SC medium lacking adenine and histidine (see Materials and Methods). We observed that co-expression of Ehg1-Cub-LV with one of the three NubG-fused nutrient permeases facilitated the growth of strain NMY51 without adenine and histidine, whereas the strain harboring Ehg1-Cub-LV alone did not (Fig. 8, compare WT and vector bars). The result suggests that Ehg1 physically interacts with these permeases in the ER. We did not observe any interactions between LV-Cub-Ehg1 and NubG-tagged amino acid permeases, suggesting the steric interference is caused by the *N*-terminal LV-Cub-tag.

Subsequently, we examined whether P17, F19, and P20, the critical Ehg1 amino acid residues for high-pressure growth, were also required for physical interactions with the nutrient permeases. The F19A or P17A-P20A mutation was created in Ehg1-Cub-LV. We observed that the mutations considerably eliminated the Ehg1–Fur4 interaction, suggesting that F19A and P17A-P20A mediate the interaction of Ehg1 with Fur4 (Fig. 8). However, the P17A-P20A but not F19A mutation abolished the Ehg1–Bap2 interaction, and the effects of the mutations were insignificant in the Ehg1–Hip1 interaction. The result suggested that the dependency on the *N*-terminal amino acid residues varied among interactions with nutrient pemeases. We confirmed that the F19A and P17A-P20A mutations in Ehg1-Cub-LV did not measurably alter the levels of NubG-tagged nutrient permeases (data not shown).

## Discussion

Our comparative and quantitative survey of previously obtained high-pressure and low-temperature sensitive mutants indicated a clear link between nutrient uptake and high-pressure growth by mutants that lacked mutually related poorly characterized genes, i.e., *EHG1/MAY24/YPR153W, MTC2*, *MTC4*, *MTC6*, *DLT1*, and *CSF1*. The extents to which plasmids for nutrient prototrophies allowed high-pressure growth were comparable in these mutants, thereby suggesting that the six genes work in the same pathway for promoting nutrient uptake. Our results demonstrated that *EHG1*, which was previously shown to have a genetic interaction profile similar to the *MTC* annotated yeast genes *MTC2* and *MTC4*^31^, encodes a novel ER membrane protein and has a critical role in maintaining nutrient permeases when cells are exposed to high pressure. In our unpublished observation, GFP-tagged Mtc6 and Dlt1 were also detected in the ER membrane upon overexpression although these proteins cannot be visualized by fluorescence microscopy at endogenous levels with a centromere-based plasmid. Therefore, we suggest that Ehg1, Mtc6 and Dlt1 might form a protein complex in the ER to exert the functionality on nutrient permeases as a novel chaperone at high pressure.

In contrast to the secondary structure predictions, Ehg1 appeared to be an ER peripheral membrane protein lacking TMDs. Therefore, we expected that the treatment of the membrane with 6 M urea could dissociate Ehg1 from the ER. However, we observed only a small amount of Ehg1 in the soluble fraction, whereas a large portion of this protein remained in the ER membrane (Fig. S2a). It was substantially solubilized with 1% Triton X-100. We speculated that Ehg1 might undergo lipid modification (palmitoylation) and become anchored onto the ER membrane^56^. To verify this hypothesis, we checked if a mutant form of Ehg1 carrying cysteine-to-glycine substitutions (C119/120 G, palmitoylation deficiency) in the HR3 could be dissociated by the urea treatment. Although the treatment slightly increased the level of Ehg1^C119/120^ in the soluble fraction, however, a substantial amount of Ehg1^C119/120^ still remained in the ER membrane (Fig. S2b). Additionally, Ehg1^C119/120^-GFP clearly localized to the ER membrane (Fig. S2c). Therefore, Ehg1 is unlikely to undergo palmitoylation to be anchored to the ER membrane. Further experiments are necessary to elucidate the mechanism underlying the stable ER association of Ehg1.

Our present yeast two-hybrid analysis suggests that Ehg1 physically interacts with nutrient permeases through its long *N*-terminal cytoplasmic tail. Therefore, we propose the hypothesis that Ehg1 might facilitate the accurate folding of nutrient permeases in the ER as a chaperone, thereby conferring resistance to the mechanical damage caused by high-pressure perturbation. High pressure would have adverse impacts on protein conformation in the ER membrane because of the stiffening effect of the lipid bilayer. Additionally, high pressure favors unfolded states of proteins because the loss of internal cavities and enhanced hydration of hydrophobic amino acid residues associated with protein unfolding are always accompanied by negative volume changes^57–61^. We assume that Ehg1 mediates subtle but important fine tuning effects on the conformation of permeases in the ER. It is particularly interesting that *MTC2* and *MTC6* have a genetic interaction with *SHR3*^62^. Shr3 is an ER packaging chaperone that plays a critical role in enabling the general amino acid permease Gap1 to fold and attain the correct structure required for functional expression in the plasma membrane^38^. In the absence of Shr3, Gap1 accumulates in the ER despite the correct insertion of the 12 TMDs. It is likely that Mtc2, Mtc6, and Ehg1 help Shr3 to increase the population of correctly folded nutrient permeases, which are stable under high-pressure perturbation. High pressure would have effects on protein conformation analogous to hydrophobic or amphipathic denaturants. Therefore, Ehg1 and Mtc proteins may have a role to confer robustness on nutrient permeases when cells are exposed to toxic chemicals in natural environments.

In yeast cells, large parts of ER called cortical ER are closely associated with the plasma membrane^49^. The average distance between the cortical ER and the plasma membrane is known to be 33 nm, and ribosomes are excluded from the cortical ER surface adjacent to the plasma membrane due to the close association^63^. Thus, we also consider the possibility that Ehg1 that resides in the cortical ER might prevent the plasma membrane permeases from pressure-induced unfolding with interactions through the long *N*-terminal tail.

The *mtc*∆ mutations are known to aggravate the mutant phenotype associated with the *cdc13-1*, mutation where the maintenance of telomere capping is defective at a restrictive temperature of 30 °C^37^. According to our supplementary results, plasmid-borne nutrient prototrophies (*TRP1*, *LEU2*, *HIS3*, and *URA3*) failed to restore normal growth at 30 °C in the *cdc13-1* mutant (W303 strain background), which suggests that the high-temperature sensitivity is not related to nutrient availability (Fig. S3a). When examining the effects of nutrient prototrophies on growth of the *cdc13-1* mutant at 25 MPa and 21 °C (permissive temperature), we unexpectedly found that the original auxotrophic *cdc13-1* (*trp1 leu2 his3 ura3 ade2*) mutant exhibited a substantial ability for growth at high pressure, although the parental wild-type strain W303 was pressure-sensitive (Fig. S3b). Thus, it is possible that the *cdc13-1* mutation causes the accumulation of Tat2 due to defects in Rsp5-dependent ubiquitination or en route to the vacuole for the degradation of Tat2^22,64^. Nutrient prototrophies did not enhance the growth ability of the *cdc13-1* mutant at 25 MPa (Fig. S3b). Therefore, high pressure is unlikely to elicit telomere capping defects to impair *CDC13*, which leads to synthetic fitness defects with the *ehg1*∆, *mtc2*∆, *mtc4*∆, and *mtc6*∆ mutations. Instead, high pressure is likely to compromise the transport activity of nutrient permeases in the absence of *EHG1* or one of the *MTC* genes under high pressure.

The *ehg1*∆ mutant and the other mutants in the MTC pathway also exhibited low-temperature sensitivity. Nutrient prototrophies efficiently restored high-pressure growth but only moderately restored low-temperature growth in these mutants although there was a positive correlation between the growth capacities (Table 1). This observation clearly indicates that the effects of high pressure and low temperature can be quantitatively discriminated with respect to the influence of the *MTC* genes on nutrient availability, thereby suggesting that they have another role in facilitating cell growth at low temperatures. Thus, it would be worthwhile examining the effects of *MTC* deletions on the activities of other classes of plasma membrane proteins, such as hexose or ammonium ion transporters, under low temperature.

In the present global survey, we also found that the high-pressure growth of some ergosterol biosynthetic mutants was restored by nutrient prototrophies (Table 1). It is known that the *erg6*∆ mutation causes missorting of tryptophan permease Tat2 to the vacuole, and therefore the *erg6*∆*trp1* mutant fails to grow in a medium with low concentration of tryptophan^65^. The *erg2*∆ mutation also promotes vacuolar degradation of Tat2^66^. Similarly, the cell surface delivery of nutrient permeases other than Tat2 is also likely to be attenuated in the *erg* mutants, and thus the nutrient supplies would be further limited under high pressure and low temperature.

The conferring of nutrient prototrophies rationalizes the restoration of the mutant phenotypes associated with the attenuation of nutrient permeases by high pressure. However, more than 50 of the high-pressure sensitive mutants could not be rescued by nutrient prototrophies, thereby indicating that various biological processes were accompanied by dynamic structural changes. The use of genetic databases and the application of functional genomic screening in studies of *S. cerevisiae* can improve our fundamental understanding of the effects of high hydrostatic pressure in living cells. In studies of “piezophysiology”, we use high hydrostatic pressure as a variable to elucidate the dynamic structural changes associated with biological processes at atmospheric pressure. The insights obtained might not be directly applicable to natural yeasts occurring in the deep sea, but they could help to identify survival strategies employed in high-pressure cold environments. Physiological and biochemical studies of deep-sea yeasts combined with genetic analyses of model yeast may help to understand the physiology of mysterious deep-sea creatures.

## Materials and Methods

### Yeast strains and culture conditions

The EUROSCARF yeast deletion library (cat. no. 95400.H3, Invitrogen, Carlsbad, CA, USA) containing 4,828 haploid gene deletion mutants and the parental strain BY4742 (*MAT****α**** his3*∆1 *leu2*∆0 *lys2*∆0 *ura3*∆0; wild type), and strain BY4741 (*MAT***a** *his3*∆0 *leu2*∆0l*met15*∆0 *ura3*∆0; wild type) were used in this study^23^. Strain NMY51 [*MATa his3*∆200 *trp1-901 leu2-3,112 ade2 LYS2*::(*lexA*op)_4_-*HIS3 ura3*::(*lexA*op)_8_-*lac*Z *ade2*::(*lexA*op)_8_-*ADE2 GAL4*] (MoBiTec GmbH, Goettingen, Germany^67^) was used for the yeast two-hybrid analysis based on the split ubiquitin system. All strains are listed in Table 2. Cells were grown at 25 °C with shaking for preculture in YPD (1% Bacto yeast extract, 2% Bacto peptone, 2% D-glucose), or synthetic complete (SC, 0.67% yeast extract nitrogen base w/o amino acids, adenine sulfate 20 µg/mL, uracil 20 µg/mL, tryptophan 40 µg/mL, histidine-HCl 20 µg/mL, leucine 90 µg/mL, lysine-HCl 30 µg/mL, arginine-HCl 20 µg/mL, methionine 20 µg/mL [only for strain BY4741], tyrosine 30 µg/mL, isoleucine 30 µg/L, phenyalanine 50 µg/mL, glutamic acid 100 µg/mL, aspartic acid 100 µg/mL, threonine 200 µg/mL, serine 400 µg/mL, 2% D-glucose) medium. To select nutrient prototrophic transformants, SD (0.67% yeast extract nitrogen base w/o amino acids, 2% D-glucose) medium was used. To culture cells under high pressure or low temperature, exponentially growing cells were diluted with SC medium into the OD_600_ value of 0.1. The diluted cultures were placed in 2.2 mL sterilized tubes and the tubes were sealed with parafilms. The tubes were subject to high pressure of 25 MPa at 25 °C in a hydrostatic chamber (PV100-100, Syn-Corporation, Kyoto, Japan) or to low temperature of 0.1 MPa at 15 °C for 24 h. At the end of the culture period, the pressure was released and the apparent optical density was measured at 600 nm (OD_600ap_) using a PD-303 spectrophotometer (Apel, Kawaguchi, Japan). The OD_600_ value, which was proportional to cell density, was calculated using a conversion formula obtained by a polynomial approximation using the spectrophotometer.

$${{\rm{OD}}}_{600}=0.0043{{\rm{A}}}^{4}-0.0168{{\rm{A}}}^{3}+0.1042{{\rm{A}}}^{2}+0.9269{\rm{A}}+0.0099$$

where A is an apparent OD_600_ (OD_600ap_) value measured without appropriate dilution. For example, the OD_600ap_ values of 0.5, 1.0, 2.0 and 3.0 are comparable to the OD_600_ values of 0.5, 1.0, 2.2 and 3.6, respectively, and are comparable to 8.25 × 10^6^, 1.65 × 10^7^, 3.3 × 10^7^, and 5.94 × 10^7^ cells/mL in our experiments. The cell density of the culture was determined using a hemocytometer.

**Table 2:** Strains used in this study.

Strain	Genotype	Source
BY4741	*MAT***a** *his3*Δ1 *leu2*Δ0 *met15*Δ0 *ura3*Δ0	^75^
BY4742	*MAT****α**** his3*Δ1 *leu2*Δ0 *lys2*Δ0 *ura3*Δ0	^75^
15568	*ehg1*Δ::*kanMX4* in BY4742	^23^
GKY31	*ehg1*Δ::*URA3* in BY4742	This study
SUY793	*ehg1*Δ::*HIS3* in BY4742	This study
14948	*mtc2*Δ::*kanMX4* in BY4742	^23^
13395	*mtc4*Δ::*kanMX4* in BY4742	^23^
12845	*mtc6*Δ::*kanMX4* in BY4742	^23^
16576	*dlt1*Δ::*kanMX4* in BY4742	^23^
12698	*csf1*Δ::*kanMX4* in BY4742	^23^
12556	*bna2*∆::*kanMX4* in BY4742	^23^
14264	*bna7*∆::*kanMX4* in BY4742	^23^
12465	*npt1*∆::*kanMX4* in BY4742	^23^
GKY762	*ehg1∆*::*URA3 bna2∆*::*kanMX4* in BY4742	This study
GKY764	*ehg1*Δ::*URA3 bna7∆*::*kanMX4* in BY4742	This study
GKY765	*ehg1*Δ::*URA3 npt1∆*::*kanMX4* in BY4742	This study
TMY1491	*SEC63*-mCherry::*URA3* in BY4742	This study
NMY51	*MAT****a**** his3*Δ200 *trp1-901 leu2-3,112 ade2 LYS2::(lexAop)4-HIS3 ura3::(lexAop)8-lacZ ade2::(lexAop)8-ADE2 GAL4 LYS2::(lexAop)4-HIS3 ura3::(lexAop)8-lacZ ade2::(lexAop)8-ADE2 GAL4*	^67,76^

### Construction of plasmids and strains

The plasmids used in this study are listed in Table 3. Primers not described below are listed in the Supplementary Table S1. pUA127 was used to construct plasmids expressing *C*-terminal 3HA-tagged fusion proteins in pRS316 (*URA3, CEN*). To construct pUA161 (*EHG1*-3HA, *URA3*, *CEN*), *EHG1* containing its intron and own promoter (p*EHG1*) was amplified using genomic DNA of strain BY4741 as a template and primers 5′- GGATTTTACGTCACCCGCCTCTTC-3′ (EHG1-F) and 5′-GGATCCGTTCTGTCCTAATGTTTGTTAAGG-3′ (EHG1-R). The resulting PCR fragment was cloned into pGEM-T Easy (Promega, Fitchburg, WI, USA) to generate pUA6. The intron was deleted by site-directed mutagenesis using PrimeSTAR Mutagenesis Basal Kit (TaKaRa Bio Inc., Shiga, Japan) to generate pUA36. The *EHG1* ORF with p*EHG1* was amplified using pUA36 as a template and primers 5′- CGGGCCCCCCCTCGAGTACGTCACCCGCCTCTTCGCTGAT-3′ and 5′- ACTCATGGTTCCCCCGGGCATAACGGAACCAACCATGGAATAACTTAG-3′. The resulting fragment was cloned into the *Xho*I-*Sma*I site of pUA127 using In-Fusion HD cloning kit (TaKaRa Bio Inc.) to generate pUA161. The *Xho*I/*Not*I digest of pUA161 was cloned into pRS426 to generate pGK15 (*EHG1*-3HA, *URA3, 2* *µ*). NEBuilder HiFi DNA assembly (New England Biolabs Japan Inc, Tokyo, Japan) was also used for plasmid constructions. To construct pUA51 (*EHG1*-GFP, *LEU2*, *2μ*), the *EHG1* ORF was amplified using pUA36 as a template and primers 5′- TTGATATCGAATTCCTGCAGTACGTCACCCGCCTCTTCGCTGAT-3′ and 5′- TGCTCACCATGGATCCCATAACGGAACCAACCATGGAATAACTTAG-3′. The resulting fragments were cloned into the *Pst*I-*Bam*HI site of pUA7 to generate the pUA51. To construct pUA264 (*EHG1*-GFP, *URA3, CEN*), the *Xho*I-*Not*I fragment containing *EHG1*-GFP in pUA51 was cloned into the *Xho*I-*Not*I site of pRS316. To construct pUA158 (5′-UTR of *EHG1*-*HIS3* marker-3′-UTR of *EHG1*), the 1.7-kb of *Not*I-*Bam*HI digest of pUA6 was cloned into pRS316 to generate pUA12. The recognition sequence of *Xba*I was newly introduced into the positions of initiation and termination codons of *EHG1*, generating pUA140. The *HIS3* marker was inserted into the *Xba*I site of pUA140 to generate pUA158. Plasmids encoding deletion mutants for the *N*-terminal domain of Ehg1 (∆2–15, ∆2–30, ∆2–45, ∆2–60, ∆2–5, ∆2–10, ∆11–20, ∆11–25, ∆11–15 and ∆16–20) were created by site-directed mutagenesis using pUA161 and primers listed in Table S1. Similarly, single or double amino acid substitutions (P11A, P17A, F19A, P20A, S21A, P25A and P17A/P20A) in Ehg1 were created in pUA161 using primers listed in Table S1. The C119/120 G mutations were created in Ehg1 by site-directed mutagenesis using pGK15 and pUA51, and primers 5′-CCACTTGGTGGCGCAGTAGTCCAAATCCTT-3′ and 5′-TACTGCGCCACCAAGTGGGACCCATGTAGA-3′ to generate pGK89 and pGK90, respectively.

**Table 3:** Plasmids used in this study.

Plasmids	Description	Source or reference
pRS313	*HIS3 CEN*	^77^
pRS315	*LEU2 CEN*	^77^
pRS316	*URA3 CEN*	^77^
pRS317	*LYS2 CEN*	^77^
pRS425	*LEU2 2μ*	^77^
pRS426	*URA3 2μ*	^77^
YCplac111	*LEU2 CEN*	^78^
pUA35	3HA driven by the *TDH3* promoter in pRS316	^72^
pYK103	*EHG1* driven by the *EHG1* promoter in YCplac111	This study
pUA161	*EHG1-*3HA driven by the *EHG1* promoter in pRS316	This study
pGK15	*EHG1-*3HA driven by the *EHG1* promoter in pRS426	This study
pUA51	*EHG1*-GFP driven by the *EHG1* promoter in pRS425	This study
pUA264	*EHG1*-GFP driven by the *EHG1* promoter in pRS316	This study
pUA268	*EHG1*-3HA Δ2-15 driven by the *EHG1* promoter in pRS316	This study
pUA269	*EHG1*-3HA Δ2-30 driven by the *EHG1* promoter in pRS316	This study
pUA270	*EHG1*-3HA Δ2-45 driven by the *EHG1* promoter in pRS316	This study
pUA271	*EHG1*-3HA Δ2-60 driven by the *EHG1* promoter in pRS316	This study
pUA333	*EHG1*-3HA Δ2-5 driven by the *EHG1* promoter in pRS316	This study
pUA334	*EHG1*-3HA Δ2-10 driven by the *EHG1* promoter in pRS316	This study
pUA335	*EHG1*-3HA Δ11-20 driven by the *EHG1* promoter in pRS316	This study
pUA336	*EHG1*–3HA Δ11-25 driven by the *EHG1* promoter in pRS316	This study
pUA348	*EHG1*–3HA Δ11-15 driven by the *EHG1* promoter in pRS316	This study
pUA349	*EHG1*-3HA Δ16-20 driven by the *EHG1* promoter in pRS316	This study
pUA408	*EHG1*-3HA P11A driven by the *EHG1* promoter in pRS316	This study
pUA353	*EHG1*–3HA P17A driven by the *EHG1* promoter in pRS316	This study
pUA364	*EHG1*-3HA F19A driven by the *EHG1* promoter in pRS316	This study
pUA354	*EHG1*-3HA P20A driven by the *EHG1* promoter in pRS316	This study
pUA365	*EHG1*-3HA S21A driven by the *EHG1* promoter in pRS316	This study
pUA355	*EHG1*-3HA P25A driven by the *EHG1* promoter in pRS316	This study
pUA389	*EHG1*-3HA P17/20A driven by the *EHG1* promoter in pRS316	This study
pGK89	*EHG1*-3HA C119/120 G driven by the *EHG1* promoter in pRS426	This study
pUA288	*EHG1*-GFP Δ2-15 driven by the *EHG1* promoter in pRS425	This study
pUA289	*EHG1*-GFP Δ2-30 driven by the *EHG1* promoter in pRS425	This study
pUA290	*EHG1*-GFP Δ2-45 driven by the *EHG1* promoter in pRS425	This study
pUA291	*EHG1*-GFP Δ2-60 driven by the *EHG1* promoter in pRS425	This study
pUM54	*EHG1*-GFP P11A driven by the *EHG1* promoter in pRS425	This study
pUM48	*EHG1*-GFP P17A driven by the *EHG1* promoter in pRS425	This study
pUM51	*EHG1*-GFP F19A driven by the *EHG1* promoter in pRS425	This study
pUM49	*EHG1*-GFP P20A driven by the *EHG1* promoter in pRS425	This study
pUM52	*EHG1*-GFP S21A driven by the *EHG1* promoter in pRS425	This study
pUM50	*EHG1*-GFP P25A driven by the *EHG1* promoter in pRS425	This study
pUM53	*EHG1*-GFP P17/20A driven by the *EHG1* promoter in pRS425	This study
pGK90	*EHG1*-GFP C119/120 G driven by the *EHG1* promoter in pRS425	This study
pMI127	3FLAG driven by the *TDH3* promoter in pRS316	This study
pYK104	*EHG1*-3FLAG driven by the *EHG1* promoter in pRS316	This study
pBT3-N	*LEU2 CEN*	^67,76^
pBT3-C	*LEU2 CEN*	^67,76^
pPR3-N	*TRP1 2 µ*	^67,76^
pCCW-Alg5	Alg5-Cub- LexA-VP16 driven by the *CYC1* promoter in pBT3-N	^67,76^
pAI-Alg5	Alg5-HA-NubI driven by the *ADH1* promoter in pPR3-C, *TRP1 2 µ*	^67,76^
pDL2-Alg5	Alg5-HA-NubG driven by the *ADH1* promoter in pPR3-C, *TRP1 2 µ*	^67,76^
pUA159	LexA-VP16-Cub-EHG1 driven by the *CYC1* promoter in pBT3-N	This study
pUA160	*EHG1*-Cub-LexA-VP16 driven by the *CYC1* promoter in pBT3-C	This study
pUA392	*EHG1* ∆76-139-Cub-LexA-VP16 driven by the *CYC1* promoter in pBT3-C	This study
pUA393	*EHG1* ∆109-139-Cub-LexA-VP16 driven by the *CYC1* promoter in pBT3-C	This study
pBT3-C-EHG1-P17,20 A	*EHG1*-P17,20A-Cub-LexA-VP16 driven by the *CYC1* promoter in pBT3-C	This study
pBT3-C-EHG1-F19A	*EHG1*-F19A-Cub-LexA-VP16 driven by the *CYC1* promoter in pBT3-C	This study
pPR3-N-3HA-HIP1	3HA-HIP1-NubG driven by the *ADH1* promoter in pPR3-C, *TRP1 2 µ*	This study
pPR3-N-3HA-BAP2	3HA-BAP2-NubG driven by the *ADH1* promoter in pPR3-C, *TRP1 2 µ*	This study
pPR3-N-FUR4-3HA	*FUR4*-3HA-NubG driven by the *ADH1* promoter in pPR3-C, *TRP1 2μ*	This study
pGK79	3HA-HIP1 driven by the *HIP1* promoter in pRS316	This study
pGK80	3HA-*HIP1* driven by the *HIP1* promoter in YCplac111	This study
pGK81	3HA-*HIP1* driven by the *HIP1* promoter in pRS425	This study
pYU65	3HA-BAP2 driven by the *BAP2* promoter in pRS316	^68^
pYU14	3HA-BAP2 driven by the *TDH3* promoter in pRS316	This study
pCA1	3HA-*BAP2* driven by the *BAP2* promoter in pRS313	This study
pGK72	*FUR4*-3HA driven by the *FUR4* promoter in pRS316	This study
pYK1	*FUR4*-3HA driven by the *FUR4* promoter in pRS425	This study
pYK3	*FUR4*-3HA driven by the *FUR4* promoter in YCplac111	This study

Plasmids used for the split-ubiquitin-based yeast two-hybrid system were generated as follows. To construct pUA159 (LexA (L)-VP16 (V)-Cub-*EHG1*, *LEU2*, *CEN*) and pUA160 (*EHG1*-LV-Cub, *LEU2*, *CEN*), the *EHG1* ORF was amplified using pUA36 as a template and primers set-1 (5′- TTGATATCGAATTCCTGCAGGAGATCATTCGTAACAAATAACGATATACCTG-3′ and 5′- TAGCTACTTACCATGGTCACATAACGGAACCAACCATGGAA-3′) and primer set-2 (5′- CACACACTAATCTAGAATGAGATCATTCGTAACAAATAACGATATACC-3′ and 5′- CGGTATCGATAAGCTTATAACGGAACCAACCATGGAATAACTTAG-3′), respectively. The resulting fragment was cloned into the *Pst*I-*Nco*I site of pBT3-N (MoBiTec GmbH) or the *Xba*I-*Hind*III site of pBT3-C (MoBiTec GmbH) to generate pUA159 and pUA160, respectively. To construct pPR3-N-FUR4-3HA, the ORF of *FUR4*-3HA was amplified using pYK3 as a template and primers 5′- TGGCCATTACGGCCCGGGAAATGCCAGACAATCTATCATT -3′ and 5′- GACATGTTTTTTCCCGGGTTATCTAGAAGCGTAATCTGGA -3′. To construct pPR3-N-3HA-HIP1, the ORF of 3HA-*HIP1* was amplified using pGK80 as a template and primers 5′- TGGCCATTACGGCCCGGGAAATGAGTTACCCATACGATGT-3′ and 5′-GACATGTTTTTTCCCGGGTTAACACCAGAAATGGAACT-3′. To construct pPR3-N-3HA-BAP2, the ORF of 3HA-*BAP2* was amplified using pYU65 as a template and primers 5′- TGGCCATTACGGCCCGGGAAATGAGTTACCCATACGATGT-3′ and 5′- GACATGTTTTTTCCCGGGTTAACACCAGAAATGATAAG-3′. Each DNA fragment was inserted into the *Sma*I site of pPR3-N.

To construct pYK1 (*FUR4-3HA, LEU2, 2μ*), *FUR4* and its own promoter (p*FUR4*) was amplified using genomic DNA of strain BY4742 as a template and primers 5′- CGGGCCCCCCCTCGAGTCTAAACCAGCATTGGGCAGCTGTC-3′ (FUR4-IFF1) and 5′-ACTCATGGTTCCCCCGGGAATGAAAGTCTTTTCGTGTTCGTGTTCGTAG-3′ (FUR4-IFR1). The resulting fragment was cloned into the *Xho*I-*Sma*I site of pUA35 to fuse a 3HA fragment to *FUR4* at the *C*-terminal end, generating pGK72. The *Kpn*I-*Not*I fragment containing *FUR4*-3HA of pGK72 was ligated into pRS425, generating pYK1. To construct pYK3 (*FUR4-*3HA*, LEU2, CEN*), the *Kpn*I/*Spe*I fragment was ligated into YCplac111.

pGK81 (3HA-*HIP1, LEU2, 2μ*) was constructed as follows. The *HIP1* ORF with its own promoter (p*HIP1*) and terminator was amplified using genomic DNA of BY4742 as a template and primers HIP1-IFF1 and HIP1-IFR1. The resulting fragment was cloned into the *Xho*I-*Xba*I site of pUA35, generating pGK69 (p*HIP1-HIP1, LEU2, CEN*). The *HIP1* ORF with its terminator was amplified using pGK69 as a template and primers HIP1-IFF4 and HIP1-IFR5. The resulting fragment was cloned into the *Xba*I-*Not*I site of pUA35, generating pGK78 (p*TDH3*-3HA-*HIP1, URA3, CEN*). The 3HA-*HIP1* was amplified using pGK69 as a template and primers HIP1-IFF5 and HIP1-IFR6. The resulting fragment was inserted into the *Xho*I-*Sma*I site of pGK78 (removal of p*TDH3*), generating pGK79 (p*HIP1*-3HA-*HIP1*, *URA3, CEN*). The DNA fragment of pGK79 digested with *Xho*I-*Not*I was inserted into pRS425, generating pGK81 (3HA-*HIP1*, *LEU2*, *2* *μ*). The DNA fragment of pGK79 digested with *Kpn*I-*Sac*I was inserted into YCplac111, generating pGK80 (3HA-*HIP1*, *LEU2*, *CEN*). To construct pYU14 (3HA-*BAP2* driven by the *TDH3* promoter in pRS316), the *Spe*I fragment of pYU13^68^ was ligated into the *Xba*I site of pUA35. The DNA fragment of pYU65 digested with *Xho*I-*Not*I was inserted into pRS313, generating pCA1 (3HA-*BAP2*, *HIS3, CEN*).

A 3FLAG fragment was amplified using pHY68 (a kind gift from Hideki Yashiroda of The University of Tokyo) as a template, and primers 5′-GAATTCCTGCAGCCCGGGGGTTCAACCATGGACTACAA-3′ and 5′-ACGTGTTTCATCTAGATCACTTGTCATCGTCATCCT-3′. The resulting fragment was inserted into the *Sma*I-*Xba*I site of pUA35, generating pMI127 (p*TDH3*-3FLAG*, URA3, CEN*). To construct pYK104 (*EHG1*-3FLAG driven by the *EHG1* promoter, *URA3, CEN*), the *EHG1* fragment was amplified using genomic DNA of strain BY4742 as a template, and primers 5′-CGGGCCCCCCCTCGAGTTCGTCTTCCTCTTCGTCT-3′ and 5′-CATGGTTGAACCCCCGGGCATAACGGAACCAACCATG-3′. The resulting fragment was inserted into the *Xho*I-*Sma*I site of pUA35 (removal of p*TDH3*).

The DNA fragment to generate the *ehg1∆*::*HIS3* mutant (SUY793) was amplified by PCR using pUA158 as a template and primers EHG1-F/EHG1-R. The *kanMX* gene was replaced by the *URA3* gene in the *ehg1*∆::kanMX4 mutant to generate the *ehg1∆*::*URA3* mutant (GKY31). The *ehg1∆*::*URA3* DNA fragments were amplified by PCR using the genomic DNA from strain GKY31 and primers EHG1-F and EHG1-R. The result fragments were used to transform the *bna2*∆::*KanMX4, bna7*∆::*KanMX4*, and *npt1*∆::*KanMX4* mutants to generate double mutants, *ehg1∆*::*URA3 bna2*∆::*KanMX4* (GKY762)*, ehg1∆*::*URA3 bna7*∆::*KanMX4* (GKY764), and *ehg1∆*::*URA3 npt1*∆::*KanMX4* (GKY765), respectively.

A PCR-based genomic tagging was carried out to generate a *C*-terminally mCherry-tagged *SEC63* strain (TMY1491)^69^. A DNA fragment was amplified using primers 5′-ATACTGATATCGATACGGATACAGAAGCTGAAGATGATGAATCACCAGAACGGATCCCCGGGTTAATTAA-3′ and 5′-TTTTTTTGGTTTTGCTTTGTATACACATGTATCTATTTTTATAAAGATGAGAATTCGAGCTCGTTTAAAC-3′, and pJT601 (mCherry, *URA3*, kind gift from Jiro Toshima) as a template to transform strain BY4742.

### ***In vitro*** COPII vesicle budding assay

Purification of COPII coat components Sar1, Sec23/24, and Sec13/31, and the vesicle budding assay were performed as previously described^70^. Microsomal membranes were prepared from yeast cells expressing Ehg1-3HA present in a high-copy plasmid. Frozen microsomal membranes were thawed and washed once with 0.5 M NaCl in buffer 88 (B88, 20 mM HEPES, 150 mM KCl, 5 mM Mg (CH_3_COO)_2_, 250 mM Sorbitol) and then twice with B88 containing protease inhibitors. The 200-µl reaction mixtures containing washed membranes (250 µg/mL), ATP plus ATP regeneration system, and GTP were incubated for 30 min at 25 °C in the presence or absence of the purified COPII coat components. After the incubation, aliquots representing the total membranes were transferred to fresh tubes. The remaining reactions were centrifuged at 10,000 × *g* for 5 min to separate the middle speed supernatant (MSS), which contained generated COPII vesicles from the heavy donor membranes. The MSS was further centrifuged at 100,000 × *g* for 1 h to collect the COPII vesicles as pellets. The total membrane fractions and COPII pellets were analyzed using SDS-PAGE and immunoblotting with anti-HA monoclonal antibody (Millipore Sigma, St. Louis, MO, USA). Additionally, Erv46 and Sec61 were considered as positive and negative controls, respectively, to determine the efficiency of the budding reaction. Signals were visualized and quantified using the LI-COR Odyssey system (LI-COR, Inc., Nebraska, USA). A percentage of each protein incorporated in the COPII vesicle fraction was compared with the total amount of each protein present in the reaction mixture. The values were plotted as packaging efficiency. Each set of reactions was performed in triplicates. Anti-Erv46 antiserum was a kind gift from Charles Barlowe (Dartmouth Medical School, USA). Anti-Sec61 antiserum was generated by injecting rabbits with a chemically synthesized Sec61 peptide coupled with keyhole limpet hemocyanin as previously reported^71^.

### Preparation of the P13 membrane fractions and subcellular fractionation

Whole cell extracts were prepared essentially as described previously^22^. Cells (1.65 × 10^8^) were collected by centrifugation, washed twice with 10 mM NaN_3_-10mM NaF, and washed once in Buffer A (50 mM Tris-HCl, pH 7.5, 5 mM EDTA, 10 mM NaN_3_). The cells were suspended with Buffer A containing 1 × Complete™ protease inhibitor mixture (EDTA-free, Roche Diagnostics, Basel, Switzerland) and mixed vigorously with glass beads. After removal of cell debris by a 5 min centrifugation at 900 × *g* at 4 °C, the supernatant (whole cell extracts) was centrifuged at 13,000 × *g* for 10 min to collect P13 membrane fractions that contained more than 90% of the plasma membrane. The precipitates were treated with 4% SDS and 5% 2-mercaptoethanol for 10 min at 37 °C. Protein concentrations were determined using a Bio-Rad Protein Assay (Bio-Rad Laboratories, Inc., Hercules, CA, USA) for equal loading of samples in SDS-PAGE.

Western blots were performed on the P13 membrane fractions as described previously using anti-HA monoclonal antibody (Medical and Biological Laboratories Co. Ltd., Nagoya, Japan), anti-Pma1 polyclonal antibody^72^, and horseradish peroxidase-conjugated anti-mouse IgG antibody (GE Healthcare, Piscataway, NJ, USA). The chemiluminescence signals were detected in an ImageQuant LAS4000 mini (GE Healthcare Life Sciences, Piscataway, NJ, USA)

Cells were also fractionated by centrifugation on a sucrose density gradient to separate the cellular membranes as described previously^68^. The cells were suspended in 50 mM Tris-HCl (pH 7.5)–1.2 M sorbitol and treated with 50 µg/mL Zymolyase 100 T for 30 min at 30 °C to obtain spheroplasts. The spheroplasts were collected by centrifugationand were homogenized wh a 27-gauge needle 6 times. After removing the unbroken cells by centrifugation, the cell lysate was placed on a sucrose density gradient (30, 45, 50, 55, and 60%) for centrifugation at 256,000 ×*g* for 5 h. Eleven fractions were collected from the top, and the proteins were treated with 5% SDS–5% 2-mercaptoethanol at 37 °C for 10 min to denature the proteins. Western blots were performed using anti-HA monoclonal antibody (Fujifilm Wako Pure Chemical Corp., Osaka, Japan), anti-Dpm1 monoclonal antibody (Invitrogen), and anti-Pma1 polyclonal antibody^72^.

### Indirect immuno-fluorescence staining

Indirect immuno-fluorescence staining was performed as described previously^73^. Briefly, cells expressing Ehg1-3HA were washed twice in 100 mM potassium phosphate buffer (pH 7.0), and fixed with 3.7% formaldehyde for 1 h. After washing twice with 0.25% NH_4_Cl in the buffer, the cells were resuspended in 1.2 M sorbitol buffer and treated with 20 µg/mL Zymolyase 100 T and 0.2% 2-mercapthoethanol for 30 min at 37 °C. After washing, the cells were resuspended in PBT (0.1% Tween 20 in the phosphate buffer), and 10 µL of the cell suspension was placed on a MAS coated slide glass (Matsunami, Tokyo, Japan), followed by serial washing with cold methanol for 6 min and cold acetone for 30 s. After drying, the cells were serially treated with anti-HA monoclonal antibody (Medical and Biological Laboratories Co. Ltd.) and Alexa 488-labeled anti-mouse rabbit polyclonal antibody (Invitrogen). The nucleus was stained with 1 µg/mL 4′,6-diamidino-2-phenylindole.

### Co-immunoprecipitation of Ehg1-3FLAG with nutrient permeases

*ehg1*∆ cells were co-transformed with pYK104 (*EHG1*-3FLAG driven by the *EHG1* promoter, *URA3 CEN*), and each one of pGK80(3HA-*HIP1* driven by the *HIP1* promoter, *LEU2 CEN*), pCA1 (3HA-*BAP2* driven by the *BAP2* promoter, *HIS3 CEN*), or pYK3 (*FUR4*-3HA driven by the *FUR4* promoter, *LEU2 CEN*). Whole cell extracts were prepared as described above and these extracts were centrifuged at 13,000 × *g* for 10 min to obtain supernatants (S13, removal of the plasma membrane). The S13 fractions were incubated with anti-FLAG M2 magnetic beads (Sigma-Aldrich, Saint Louis, MO, USA) in Buffer B (Buffer A supplemented with 150 mM NaCl and 1% Triton X-100) for 1 h at 4 °C. The beads were washed thrice with Buffer B. The bound Ehg1-3FLAG proteins were obtained by competitive elution with 100 µg/mL FLAG peptide (Sigma-Aldrich). Eluted samples were analyzed using Western blotting to detect 3HA-tagged nutrient permeases and Ehg1-3FLAG using the anti-HA monoclonal antibody and anti-FLAG monoclonal antibody (Sigma-Aldrich), respectively.

### Yeast two-hybrid system based on the split-ubiquitin mechanism

A yeast two-hybrid membrane protein system exploiting the split-ubiquitin mechanism was performed to analyze the membrane topology of Ehg1. In this system, two target membrane proteins were fused with ubiquitin molecules that were split into two halves designated as Nub (for *N*-terminal ubiquitin) and Cub (for *C*-terminal ubiquitin) on the cytoplasmic side^67,74^. Cub has been fused to the artificial transcription factor LexA-VP16. When the Nub- and Cub-LexA-VP16 (LV)-fused proteins are co-expressed within the same cell (strain NMY51), the strong affinities of the Nub and Cub portions lead to an efficient re-assembly (split-ubiquitin) with no interaction between the target proteins. The split-ubiquitin system is immediately recognized by ubiquitin-specific proteases, thereby leading to the cleavage of the polypeptide chain to release LexA-VP16. Thereafter, LexA-VP16 translocates to the nucleus where it activates *HIS3* and *ADE2*, whose activation enables the cells to grow on SC medium lacking histidine or adenine. Therefore, the expression of the reporter genes can only be induced when both Nub and Cub were located on the cytoplasmic side of the membrane. Plasmids expressing LV-Cub-Ehg1 (WT, pUA159), Ehg1-Cub-LV (WT, pUA160), or Ehg1-Cub-LV variants (pUA392 or pUA393) were used as bait vectors. A control plasmid expressing Alg5-Nub (pAI-Alg5), which is an integral ER membrane protein (*C*-terminal tail is faced to the cytoplasm)^52^, was used as a prey vector (Table 3). The cells were cultured in SC medium with or without histidine-HCl (20 µg/mL) and adenine sulfate (20 µg/mL) for 24 h starting at the OD_600_ value of 0.1.

The split-ubiquitin system was used to analyze physical interactions between Ehg1 and the nutrient permeases Hip1, Bap2, and Fur4. For this analysis, the mutated *N*-terminal half of ubiquitin NubG (I3G) was used to avoid the self-assembly of split-ubiquitin. Strain NMY51 was co-transformed with a bait (Ehg1-Cub-LV, pUA160) and a prey (nutrient permease-NubG, pPR3-N-3HA-HIP1, pPR3-N-3HA-BAP2 or pPR3-N-FUR4-3HA) plasmid (Table 3). The cells were cultured in SC medium with or without histidine-HCl (20 µg/mL) and adenine sulfate (20 µg/mL) for 24 h starting at the OD_600_ value of 0.1.

### Substrate uptake assay

L-[ring-2, 5-^3^H] histidine (MT-905, 1.38 TBq/mmol, 37 MBq/mL in ethanol: water (2: 98); Moravek Inc., Brea, CA, USA), L-[3, 4, 5-^3^H]-leucine (NET460, 3.7 TBq/mmol, 37 MBq/mL in ethanol: water (2: 98); PerkinElmer Inc., Boston, MA, USA), and [5, 6-^3^H]-uracil (MT-512, 1.52 TBq/mmol, 37 MBq/mL in water, Moravek Inc.), were used for substrate uptake assay as described previously with some modifications^72^. The wild-type, *ehg1*∆, *mtc2*∆, *mtc4*∆, *mtc6*∆, *dlt1*∆, and *csf1*∆ cells were grown in SC medium for overnight at 25 °C until the OD_600_ value of 1.0–2.0. The cells were incubated at 0.1 or 25 MPa in a hydrostatic chamber for additional 3 h after dilution of the cultures to adjust the OD_600_ value of 1.0 with fresh SC medium. After decompression, the cells were collected by centrifugation, washed twice and resuspended in the assay buffer (50 mM 2-Morpholinoethanesulfonic acid, 20 mM (NH_4_)_2_SO_4_, 2% D-glucose, pH 5.0) at a cell density of approximately 3 × 10^7^ cells/mL to perform substrate uptake assay at 0.1 MPa. Concentrations of the substrates in each assay buffer were 2 *μ*g/mL histidine HCl monohydrate (9.5 µM), 9 *μ*g/mL leucine (68.6 µM), and 2 *μ*g/mL uracil (17.8 µM) with a 1/4000 of the total volume of each [^3^H]-labeled substrate. A vacuum aspirator was used to trap the cell suspension on a GF/C glass filter at time points of 15, 30, and 60 min, followed by a wash step with 10 mL of ice-cold distilled water containing non-labeled 30 *μ*g/mL histidine HCl monohydrate, 135 *μ*g/mL leucine, or 30 *μ*g/mL uracil. The quantity of incorporated substrate was then measured using a liquid scintillation counter. In our experimental conditions, 1 DPM for [^3^H]-histidine, [^3^H]-leucine, or [^3^H]-uracil, is converted to 17.6, 138.9, or 29.4 fmol of incorporated non-labeled histidine, leucine, or uracil in the cells, respectively. Data are expressed as mean values of incorporated substrate (pmol/10^7^ cells) with standard deviations obtained from three independent experiments.

### Fluorescence microscopy

Cells expressing GFP-tagged proteins or Ehg1-3HA with the immuno-staining were imaged on a fluorescence microscope model IX70 (Olympus, Co. Ltd, Tokyo, Japan) or a confocal laser microscope model FV-3000 (Olympus, Co. Ltd).

## Supplementary information

Supplementary text and figures
