# Supplementary material for: A novel ER membrane protein Ehg1/May24 plays a critical role in maintaining multiple nutrient permeases in yeast under high-pressure perturbation
Source: Sci Rep. 2019 Dec 4;9:18341. doi: 10.1038/s41598-019-54925-1 (PMC6892922; doi:10.1038/s41598-019-54925-1)
Supplement: Supplementary file 1 — Supplementary text and figures [file 41598_2019_54925_MOESM1_ESM.pdf]

Supplementary information

A novel ER membrane protein Ehg1/May24 plays a critical role in maintaining multiple nutrient permeases in yeast under high-pressure perturbation

Goyu Kurosaka[1], Satoshi Uemura[2], Takahiro Mochizuki[1], Yuri Kozaki[1], Akiko Hozumi[1], Sayuri Suwa[1], Ryoga Ishii[1], Yusuke Kato[1], Saki Imura[1], Natsuho Ishida[1], Yoichi Noda[3], and Fumiyoshi Abe[1]*

[1]Department of Chemistry and Biological Science, College of Science and Engineering, Aoyama Gakuin University, Sagamihara, Japan; [2]Division of Medical Biochemistry, Faculty of Medicine, Tohoku Medical and Pharmaceutical University, Sendai, Japan; [3]Collaborative Research Institute for Innovative Microbiology, Department of Biotechnology, The University of Tokyo, Tokyo, Japan

*Corresponding author: Fumiyoshi Abe
5-10-1 Fuchinobe, Chuo-ku, Sagamihara 252-5258, Japan
Phone: +81-42-759-6233
Fax: +81-42-759-6511
E-mail: abef@chem.aoyama.ac.jp

Table S1. Primers used in this study.

Plasmid name	ORF	Deletion	Primer name	Sequence	Template
pUA268	Ehg1-3HA	Δ2-15	YPR153w-del2-15-F1	GTATTATGACGCCAAAATTCCCCTCA	pUA161
			YPR153W-del2-15-R1	TTGGCGTCATAATACGGCAGGAAGT	
pUA269	Ehg1-3HA	Δ2-30	YPR153w-del2-30-F1	GTATTATGTATAATACTGCATTCTTA	pUA161
			YPR153W-del2-30-R1	TATTATACATAATACGGCAGGAAGT	
pUA270	Ehg1-3HA	Δ2-45	YPR153w-del2-45-F1	GTATTATGTCGCTGTACTGGACTTTG	pUA161
			YPR153W-del2-45-R1	ACAGCGACATAATACGGCAGGAAGT	
pUA271	Ehg1-3HA	Δ2-60	YPR153w-del2-60-F1	GTATTATGGCTGGTGTGTATGCTAGC	pUA161
			YPR153W-del2-60-R1	CACCAGCCATAATACGGCAGGAAGT	
pUA333	Ehg1-3HA	Δ2-5	YPR-Delta2-5-F1	GTATTATGACAAATAACGATATACCT	pUA161
			YPR-Delta2-5-R1	TATTTGTCATAATACGGCAGGAAGT	
pUA334	Ehg1-3HA	Δ2-10	YPR-Delta2-10-F1	GTATTATGCCTGTTGGATATGTAACG	pUA161
			YPR-Delta2-10-R1	CAACAGGCATAATACGGCAGGAAGT	
pUA335	Ehg1-3HA	Δ11-20	YPR-Delta11-20-F1	ACGATATATCATTATACTGGCCCATC	pUA161
			YPR-Delta11-20-R1	ATAATGATATATCGTTATTTGTTAC	
pUA336	Ehg1-3HA	Δ11-25	YPR-Delta11-25-F1	ACGATATAATCAACAACTCGAAGTAT	pUA161
			YPR-Delta11-25-R1	TGTTGATTATATCGTTATTTGTTAC	
pUA348	Ehg1-3HA	Δ11-15	YPR153-Delta11-15-F1	CGATATAACGCCAAAATTCCCCTCA	pUA161
			YPR153-Delta11-15-R1	TTTGGCGTTATATCGTTATTTGTTAC	
pUA349	Ehg1-3HA	Δ16-20	YPR153W-Delta16-20-F1	ATATGTATCATTATACTGGCCCATC	pUA161
			YPR153W-Delta16-20-R1	TATAATGATACATATCCAACAGGTAT	
pUA408	Ehg1-3HA	P11A	YPR-P11A-F1	CGATATAGCTGTTGGATATGTAACGC	pUA161
			YPR-P11A-R1	CCAACAGCTATATCGTTATTTGTTAC	
pUA353	Ehg1-3HA	P17A	YPR-P17A-F1	TGTAACGGCAAAATTCCCCTCATTAT	pUA161
			YPR-P17A-R1	AATTTTGCCGTTACATATCCAACAGG	
pUA354	Ehg1-3HA	P20A	YPR-P20A-F1	AAAATTCGCCTCATTATACTGGCCCA	pUA161
			YPR-P20A-R1	AATGAGGCGAATTTTGGCGTTACATA	
pUA355	Ehg1-3HA	P25A	YPR-P25A-F1	ATACTGGGCCATCAACAACTCGAAGT	pUA161
			YPR-P25A-R1	TTGATGGCCCAGTATAATGAGGGGAA	
pUA364	Ehg1-3HA	F19A	YPR-F19A-F1	GCCAAAAGCCCCCTCATTATACTGGCC	pUA161
			YPR-F19A-R1	GAGGGGGCTTTTGGCGTTACATATCC	
pUA365	Ehg1-3HA	S21A	YPR-S21A-F1	ATTCCCCGCATTATACTGGCCCATCA	pUA161
			YPR-S21A-R1	TATAATGCGGGGAATTTTGGCGTTAC	
pUA389	Ehg1-3HA	P17/20A	YPR-P20A-F1	AAAATTCGCCTCATTATACTGGCCCA	pUA353
			YPR-P20A-R2	AATGAGGCGAATTTTGCCGTTACATA	
pUA392	Ehg1-Cub-LV	Δ76-139	Ypr-CLV-del76-139-F1	CTGGAAGTATAAGCTTATCGATACCG	pUA160
			Ypr-CLV-del76-139-R1	AGCTTATACTTCCAGCCTTCTTTCT	
pUA393	Ehg1-Cub-LV	Δ109-139	Ypr-CLV-del109-139-F1	GATCTGGTATAAGCTTATCGATACCG	pUA160
			Ypr-CLV-del109-139-R1	AGCTTATACCAGATCTATAGATCGC	

Supplementary Figure legends

Fig. S1. Deletions for *BNA2*, *BNA7* or *NPT1* do not restore the high-pressure growth ability in the *ehg1*Δ mutant. The wild-type strain, and single or double deletion mutants were cultured at 0.1 MPa or 25 MPa and 25°C for 24 h, starting at the OD_{600} value of 0.1. Data are represented as means and standard deviations of three independent experiments.

Fig. S2. The full-length gel image of Western blotting for Ehg1 and Ehg1$^{C119/120G}$, and localization of Ehg1$^{C119/120G}$-GFP in Fig. S2. The P100 membrane fractions were obtained from the *ehg1*Δ mutant expressing (a) Ehg1-3HA and (b) Ehg1$^{C119/120G}$-3HA after disruption of the cells with glass beads. The membrane fractions were treated with 6 M urea, or 1% Triton X-100 for 1 h at 4°C. The resulting samples were subject to centrifugation at 100,000 × g for 1 h to separate the membrane and soluble fractions. (c) The *ehg1*Δ cells expressing Ehg1-GFP or Ehg1$^{C119/120G}$-GFP (*LEU2*, *2μ*) were imaged under a confocal laser microscope

Fig. S3. Cell growth of the *cdc13-1* mutant with plasmid-borne nutrient prototrophies at restrictive temperature or high pressure (a) The wild-type strain (W303-1A, *MAT***a** *his3-11,15 leu2-3,112 ura3-1 trp1-1 can1-100 ade2-100*) and the *cdc13-1* mutant (N283-2, *cdc13-1* in W303-1A, a kind gift of Akira Matsuura of Chiba University [1]) harboring pRS314 (*TRP1, CEN*), or 4 plasmids pRS313 (*HIS3, CEN*), pRS314, pRS315 (*LEU2, CEN*) and pRS316 (*URA3, CEN*) together were cultured in SC medium under indicated conditions. (b) The wild-type strain and the *cdc13-1* mutant harboring an empty vector (*URA3*, 2μ) or YEpCDC13 (*URA3*, 2μ, a kind gift of Naoyuki Hayashi Kanazawa Gakuin University [2]) were cultured in SC medium under indicated conditions.

Fig. S4. The full-length gel image of Western blotting for Ehg1-3HA, Pma1 and Dpm1 in Fig. 3E.

Fig. S5. The full-length gel image of Western blotting for Hip1 and Pma1 in Fig. 4A.

Fig. S6. The full-length gel image of Western blotting for Bap2 and Pma1 in Fig. 4A.

Fig. S7. The full-length gel image of Western blotting for Fur4 and Pma1 in Fig. 4A. The Fur4 band at 24 h following pressurization is not shown in Fig. 4A.

Fig. S8. The full-length gel image of Western blotting for in Fig. 7 Exp.1.

Fig. S9. The full-length gel image of Western blotting for in Fig. 7 Exp.2.

References

1. Miura, A. & Matsuura, A. Phosphatase-dependent fluctuations in DNA-damage checkpoint activation at partially defective telomeres. *Biochem. Biophys. Res. Commun.* **516**, 133-137 (2019).
2. Hayashi, N. & Murakami, S. STM1, a gene which encodes a guanine quadruplex binding protein, interacts with *CDC13* in *Saccharomyces cerevisiae*. *Mol. Genet. Genomics* **267**, 806-813 (2002).

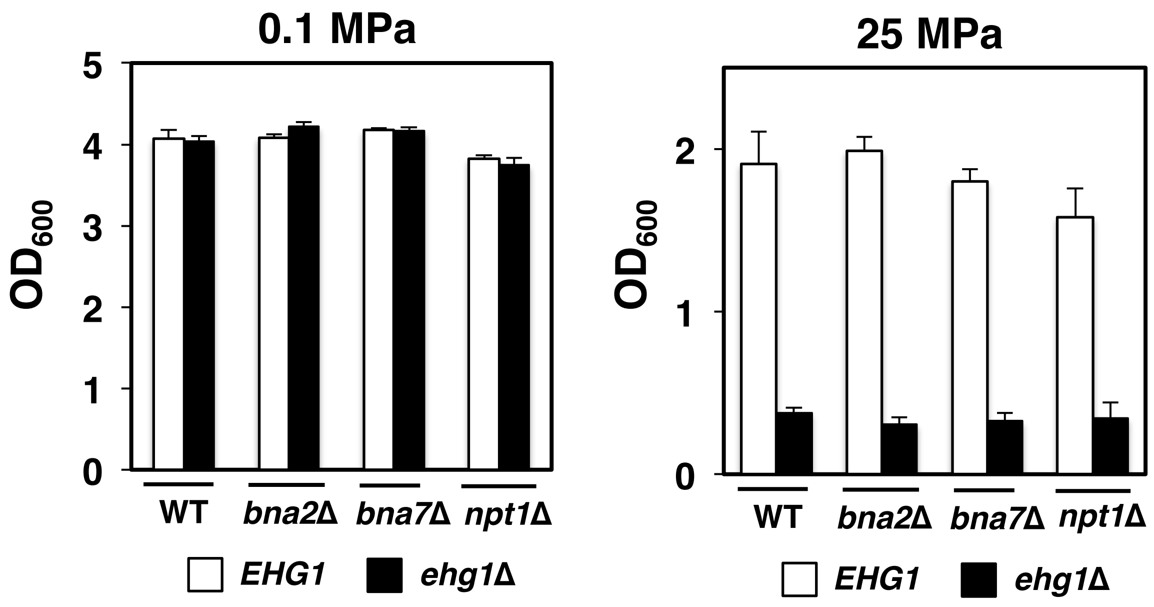

Figure S1

The full-length gel images of Western blotting for Ehg1 and Ehg1$^{C119/120G}$, and localization of Ehg1$^{C119/120G}$-GFP in Fig. S2

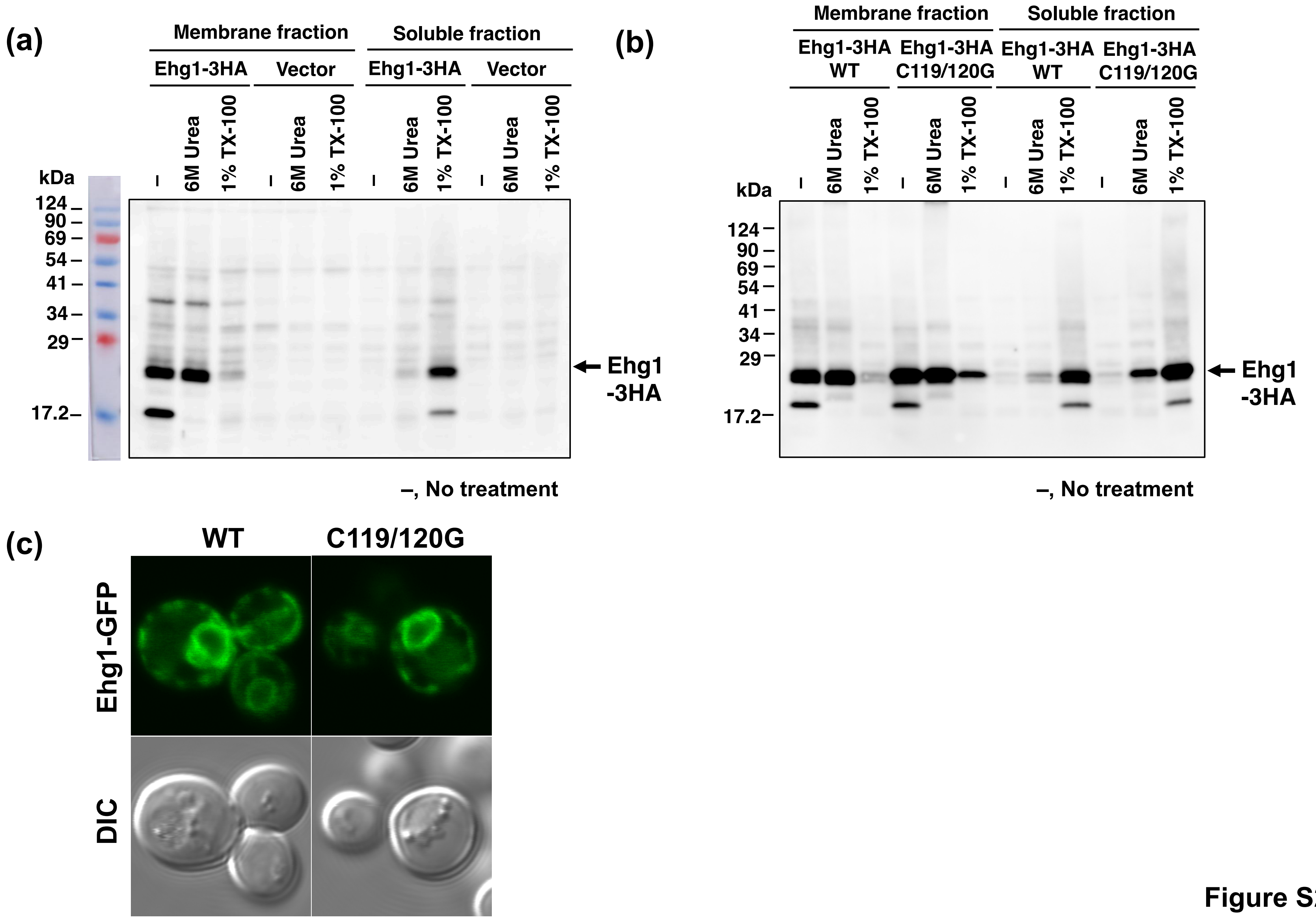

Figure S2

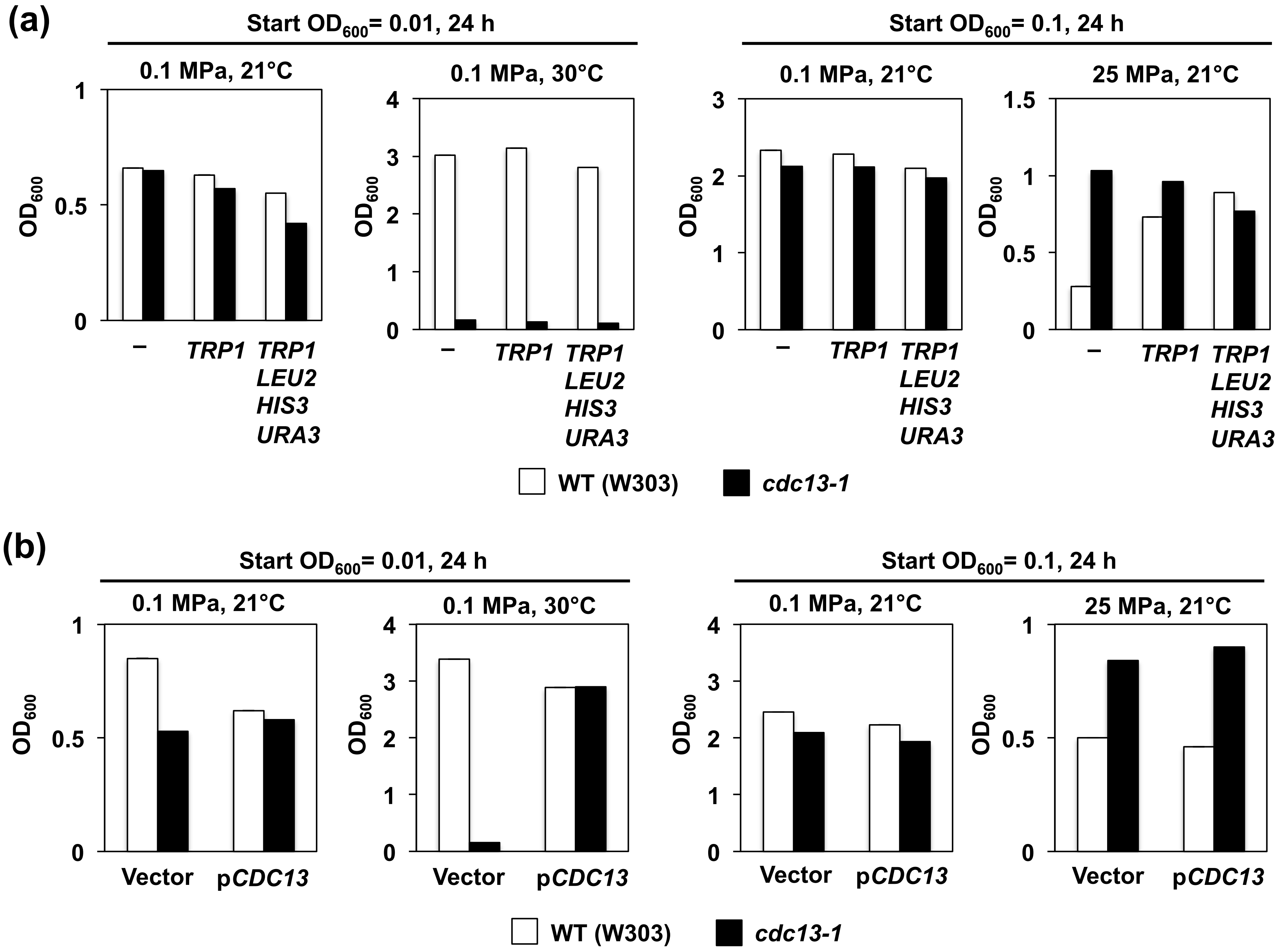
(a)
Start OD600= 0.01, 24 h
Start OD600= 0.1, 24 h
0.1 MPa, 21°C
0.1 MPa, 30°C
0.1 MPa, 21°C
25 MPa, 21°C
OD600
–
TRP1
TRP1 LEU2 HIS3 URA3
WT (W303)
cdc13-1
(b)
Start OD600= 0.01, 24 h
Start OD600= 0.1, 24 h
0.1 MPa, 21°C
0.1 MPa, 30°C
0.1 MPa, 21°C
25 MPa, 21°C
OD600
Vector
pCDC13
WT (W303)
cdc13-1

Figure S3

The full-length gel image of Western blotting for Fig. 3E

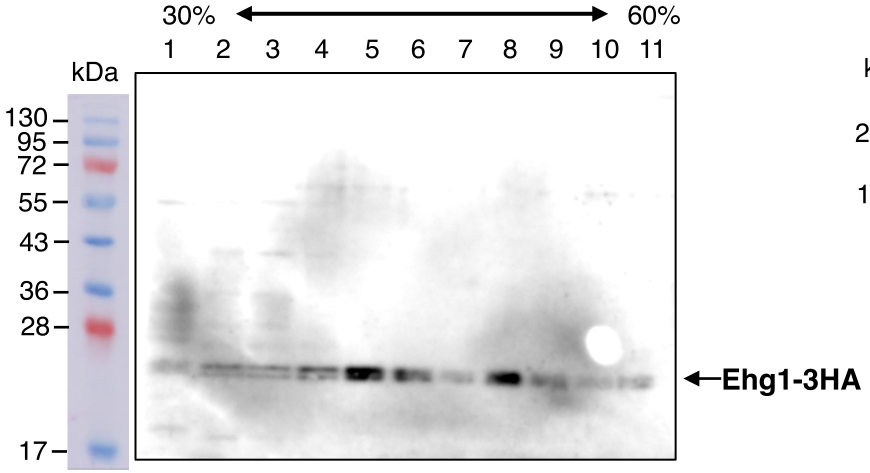

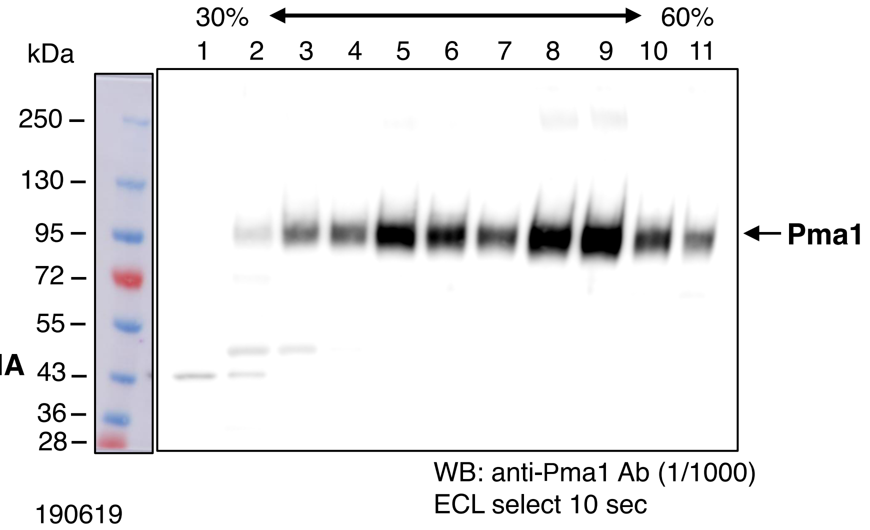

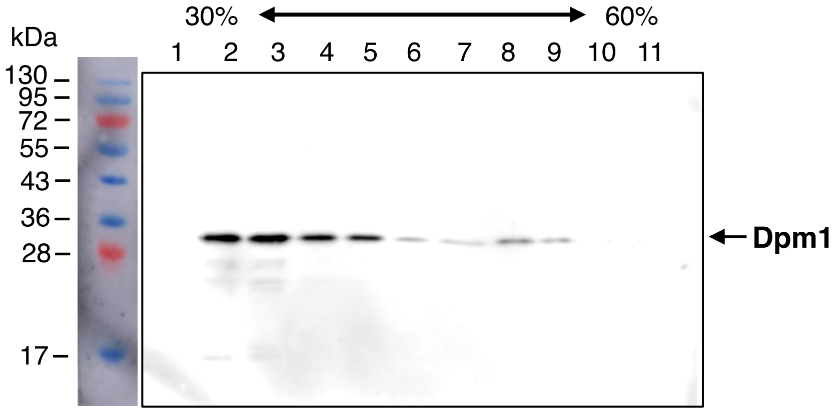

Figure S4

The full-length gel image of Western blotting for Hip1 and Pma1 in Fig. 4A

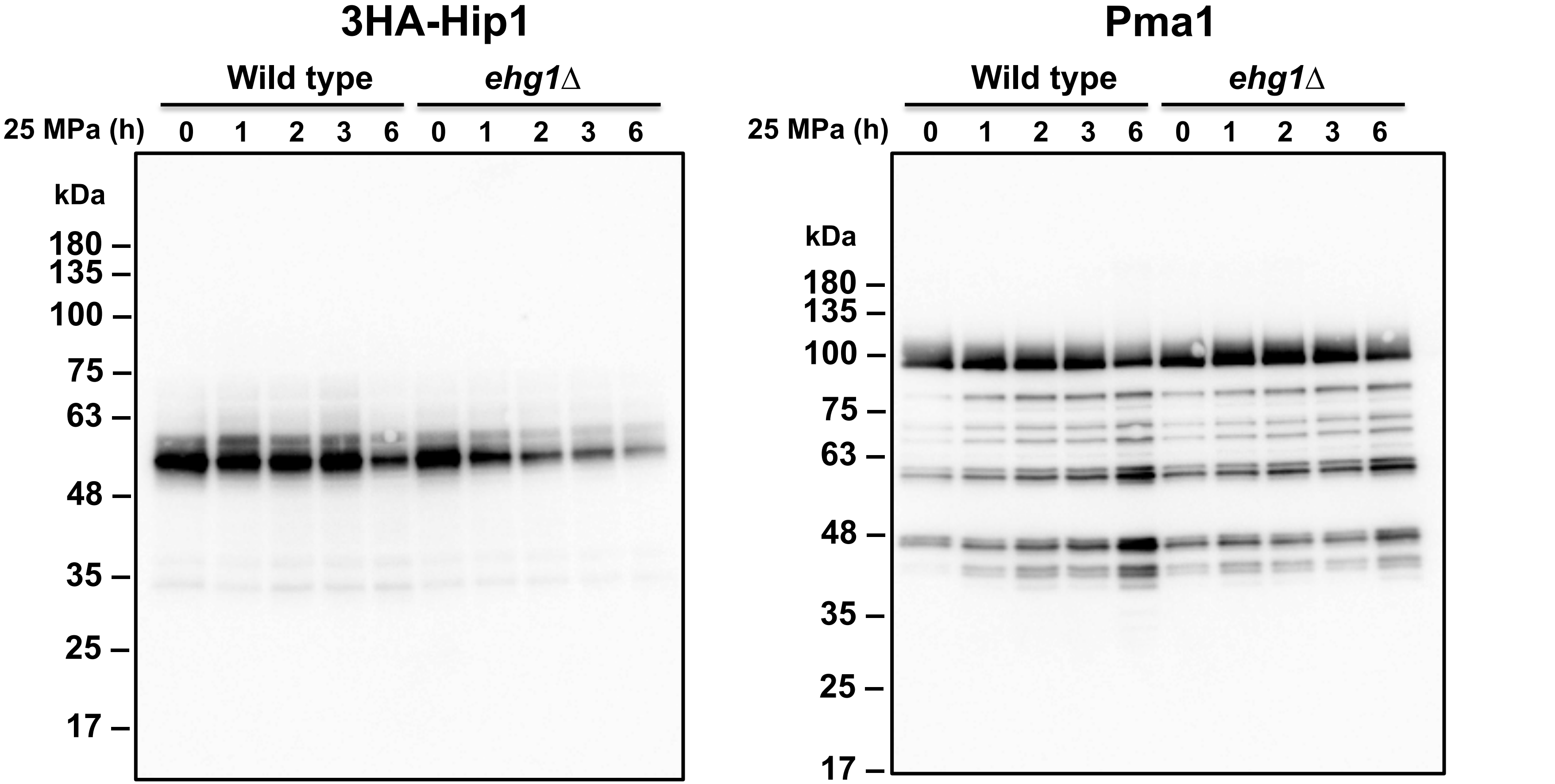

Figure S5

The full-length gel image of Western blotting for Bap2 and Pma1 in Fig. 4A

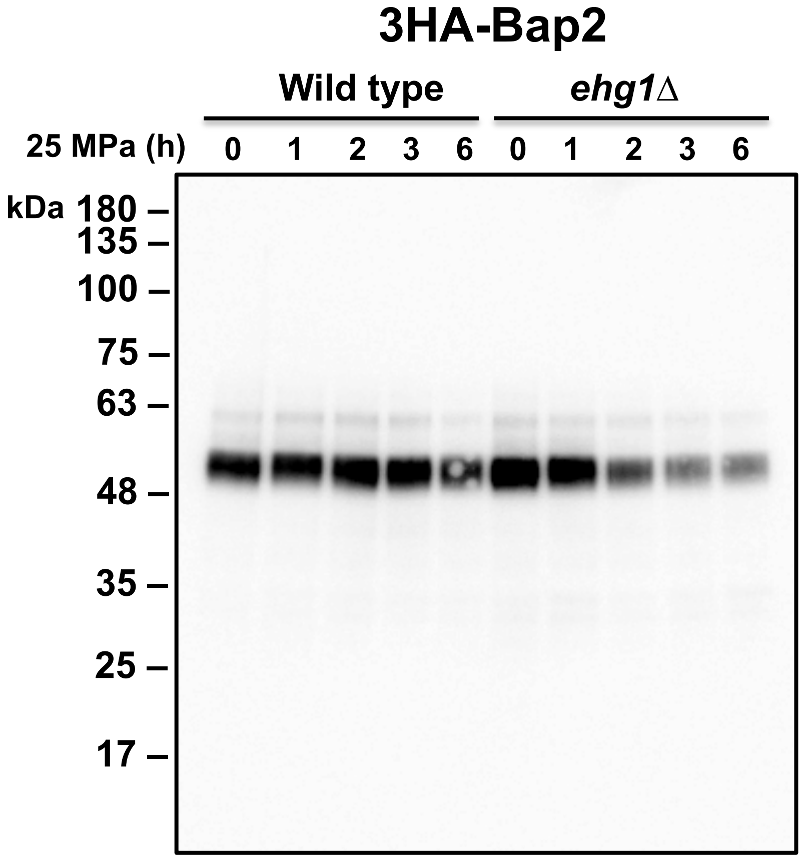

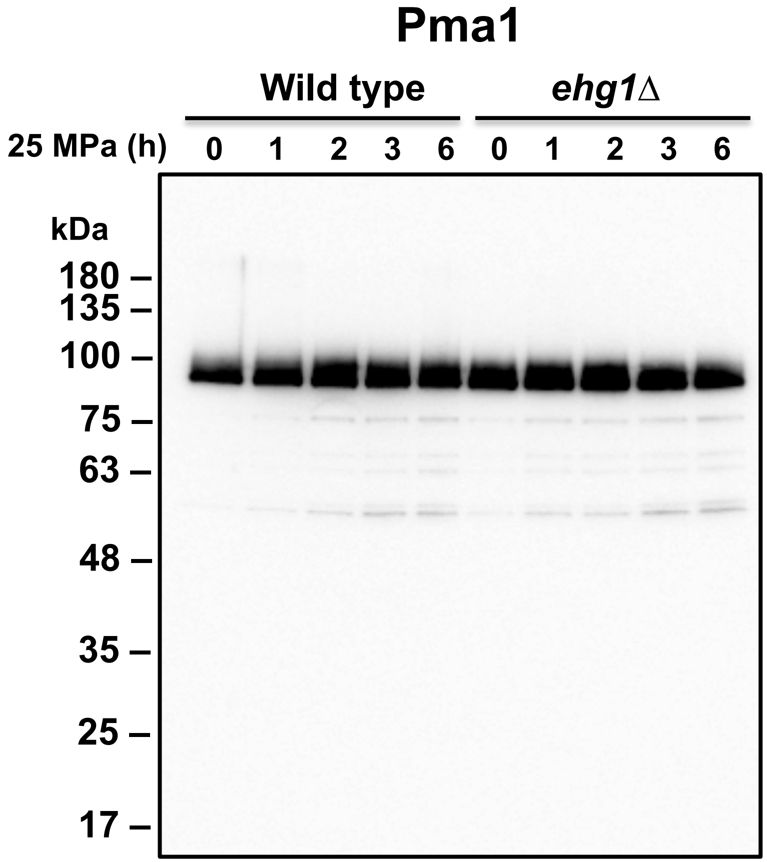

Figure S6

The full-length gel image of Western blotting for Fur4 and Pma1 in Fig. 4A

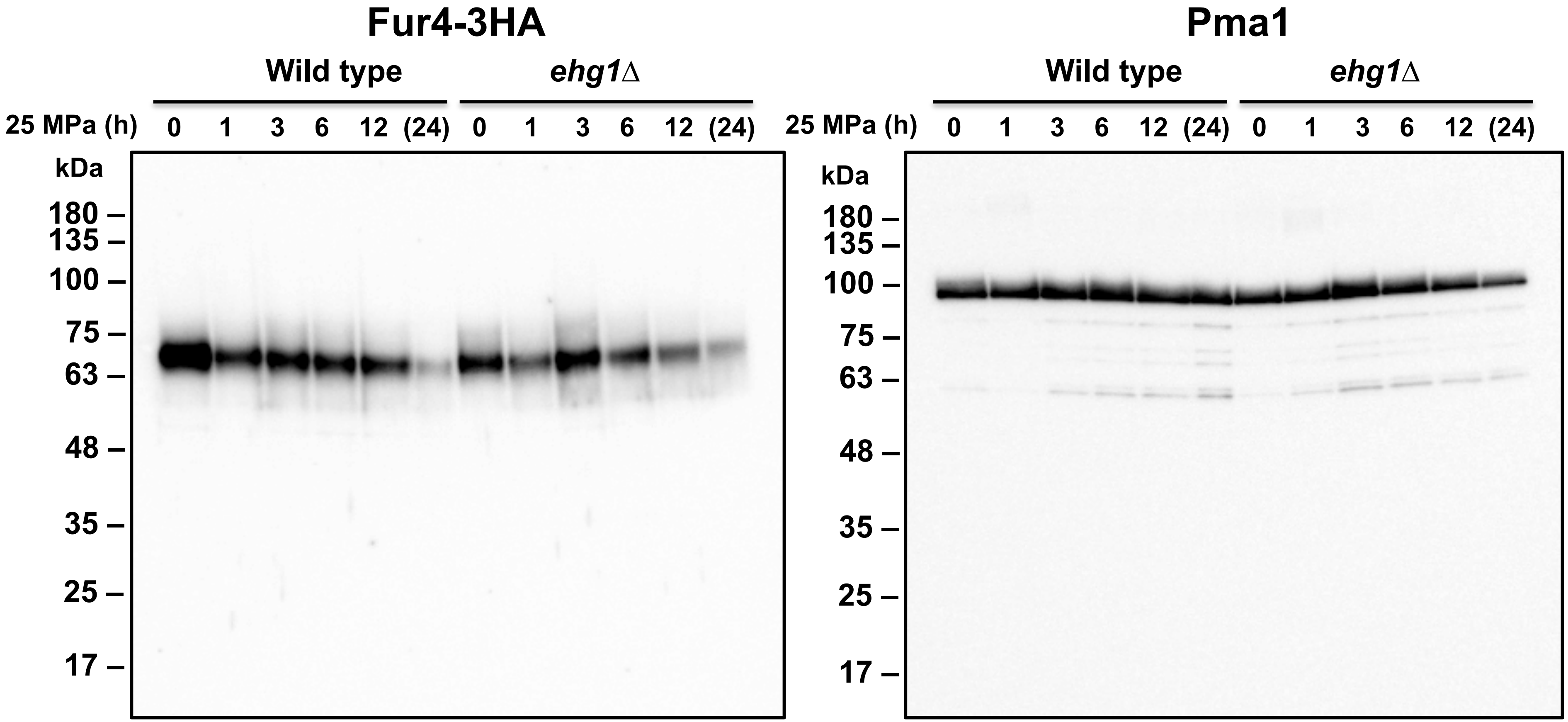

Figure S7

The full-length gel image of Western blotting for Fig. 7 Exp.1

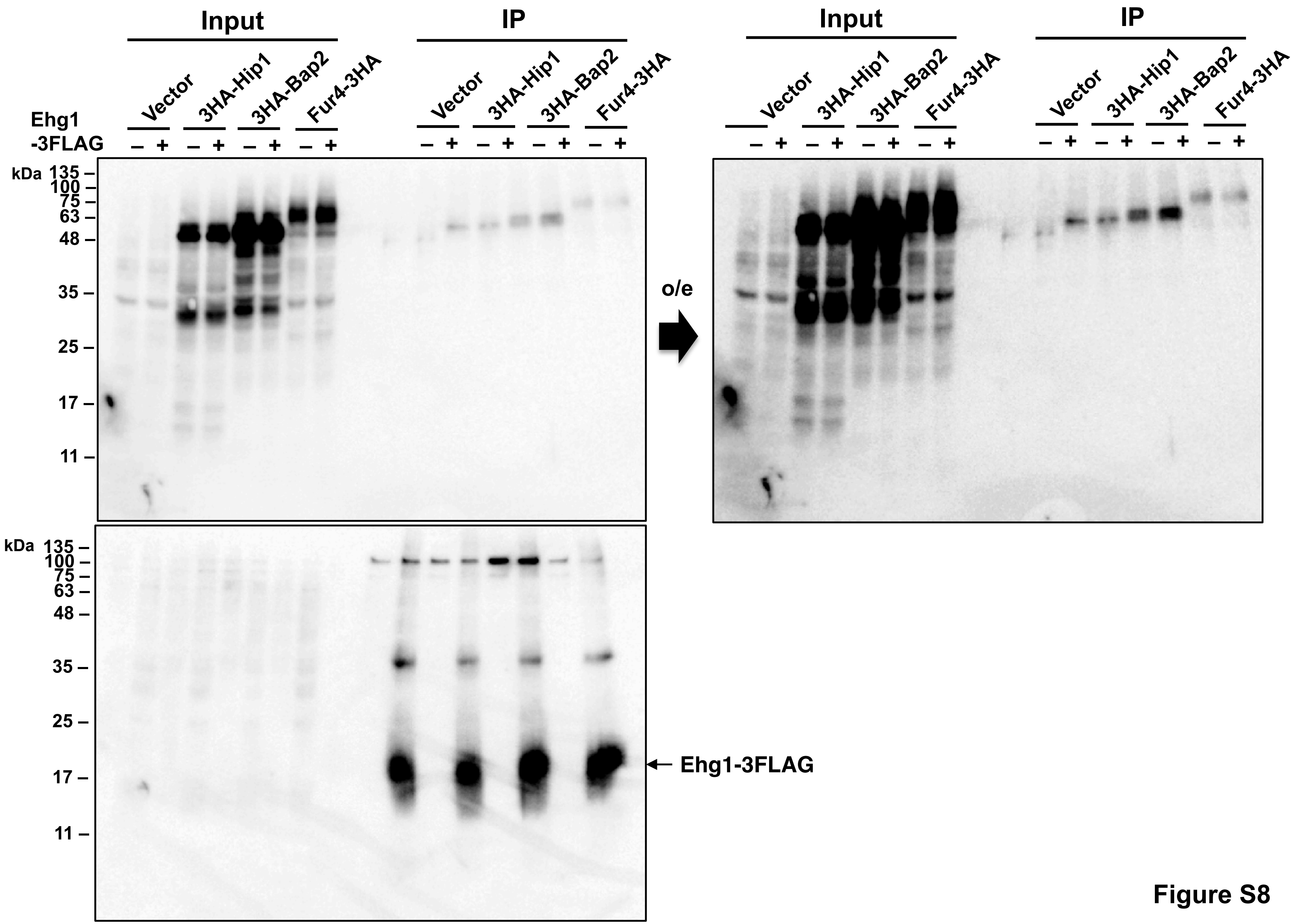

Figure S8

The full-length gel image of Western blotting for Fig. 7 Exp.2

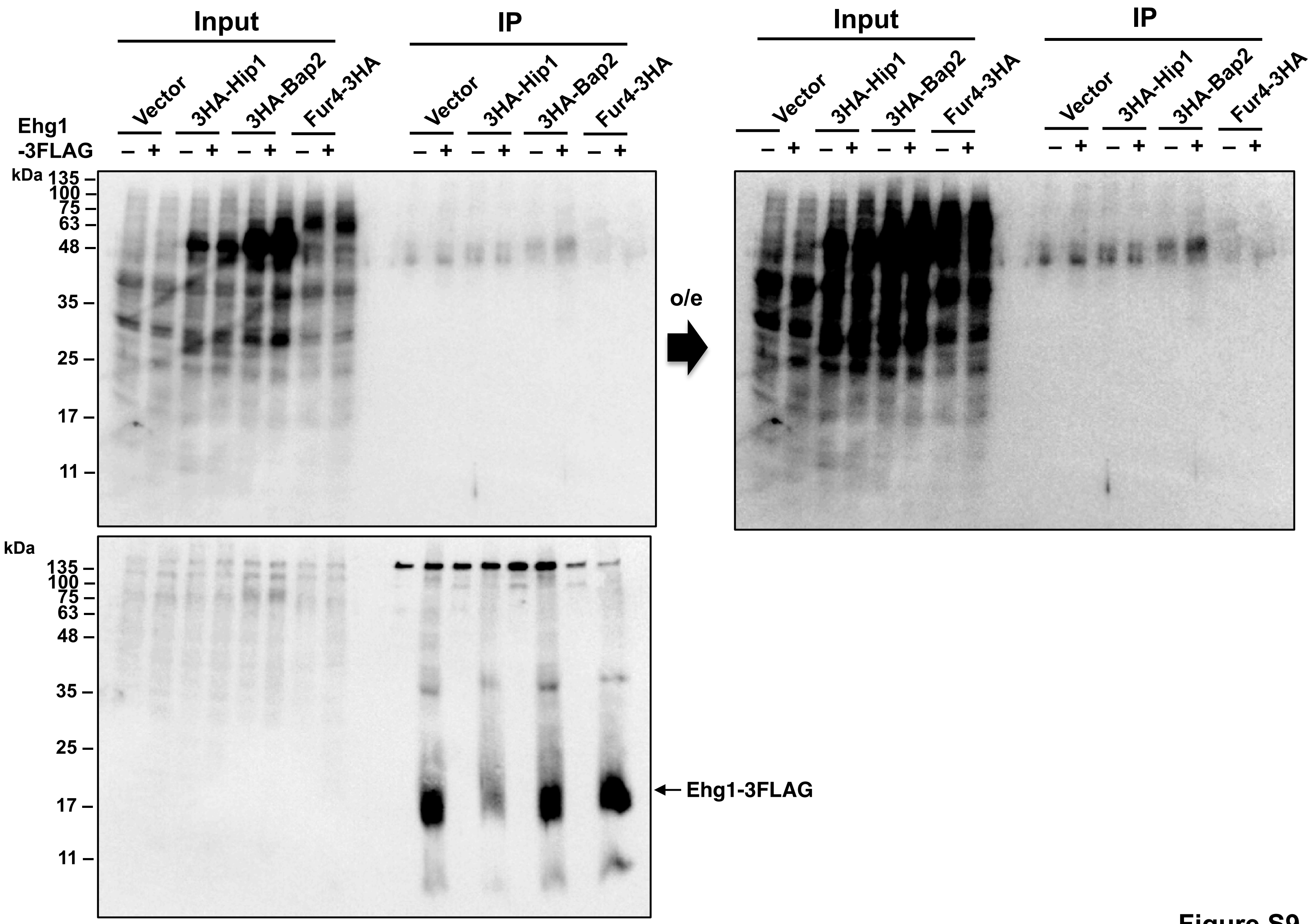

Figure S9
